# Supplementary material for: Percolation‐Driven β‐Relaxation Enables Resonant Acceleration of Crystallization in Amorphous Phase‐Change Materials
Source: Adv Mater. 2026 Sep 27;38(63):e75151. doi: 10.1002/adma.75151 (PMC13755648; doi:10.1002/adma.75151)
Supplement: Supplementary file 1 [file ADMA-38-e75151-s001.pdf]

Supplementary Information

Percolation-driven β-relaxation enables resonant control of crystallization in amorphous phase-change materials

Yu-Yao Liu[#1], Liang Gao[#1], Jun-Ying Jiang[1], Yiming Zhou[2], Jan Luebben[3], Di Zhao[2], Xiaoling Lu[4], Maximilian J. Müller[2], Ulrich Boettger[3], Jiang-Jing Wang*[2,5], Hai-Bin Yu*[1] and Shuai Wei*[6,7]

[1]*Wuhan National High Magnetic Field Center and School of Physics, Huazhong University of Science and Technology, Wuhan, Hubei 430074, China*

[2]*Institute of Physics (IA), RWTH Aachen University, Aachen, 52056, Germany.*

[3]*Institut für Werkstoffe der Elektrotechnik II (IWE II), Aachen, 52056, Germany.*

[4]*Institut für Werkstoffe der Elektrotechnik I (IWEI), Aachen, 52056, Germany.*

[5]*Center for Alloy Innovation and Design (CAID), State Key Laboratory for Mechanical Behavior of Materials, Xi'an Jiaotong University, Xi'an, 710049, China*

[6]*Department of Chemistry, Aarhus University, 8000 Aarhus C, Denmark*

[7]*CENSEMAT Center for Sustainable Energy Materials, Aarhus University, Denmark.*

1. Methods

1. 1. Simulations

Molecular dynamics simulations and dynamic mechanical spectroscopy

We use an open-source molecular dynamics (MD) simulation code, LAMMPS[1], for our all the simulations. We conduct molecular dynamics (MD) simulations using a DeepMD neural network potential developed by O. Abou El Kheir et al[2, 3] for $Ge_2Sb_2Te_5$ system. The $Ge_2Sb_2Te_5$ glass model which contains N=6912 atoms in cubic box, is obtained by continuous cooling with a cooling rate of 10^{12} K/s from 1000 K to 10 K. This model is initially equilibrated at a sufficiently high temperature and then cooled with the specified cooling rate with periodic boundary condition and the NVT ensemble (that is, a constant number of atoms, volume, and

# These authors contributed equally.

*j.wang@xjtu.edu.cn (JJW), haibinyu@hust.edu.cn (HBY), shuai.wei@chem.au.dk (SW)

temperature). Then the glassy system is heated to near its crystallization temperature at a rate of 10^{10} K/s. Configurations during heating are saved for subsequent analysis.

The MD simulation of DMS (MD-DMS) is performed during the heating process, using the initial configurations as discussed above. From 10 K to the crystallization temperature, we take a configuration at intervals of 10K for MD-DMS analysis. In MD-DMS, we apply a sinusoidal shear strain

$$\varepsilon(t) = \varepsilon_A \, sin(2\pi t/\, t_\omega) \tag{1}$$

with an oscillation period t_ω (related to frequency $f = 1/t_\omega$) and a strain amplitude ε_A, along the *x* direction of $Ge_2Sb_2Te_5$ model. The value of t_ω is 1 nanoseconds (ns). The resulting shear stress $\sigma(t)$ is computed and fitted according to

$$\sigma(t) = \sigma_0 + \sigma_A \, sin(2\pi t/\, t_w + \delta) \tag{2}$$

wherein δ is the phase angle between stress and strain. The σ_0 is a linear term and is usually small. The strain was applied by a smooth change of the box shape, whereas stresses are directly measured from each component of the pressure. The storage E' and loss E'' moduli are calculated as below:

$$E' = \frac{\sigma_A}{\varepsilon_A} cos(\delta)\text{;}\;\; E'' = \frac{\sigma_A}{\varepsilon_A} sin(\delta) \tag{3}$$

A strain amplitude $\varepsilon_A = 1\%$ is applied in all the MD-DMS simulations. Such a value of strain amplitude ensures that the deformations do not change the structure and internal energy of the models. In other words, the deformation is in the linear response regime and the dissipation-fluctuation theorem is valid. Technically, the NVT ensemble (constant number of atoms, volume and temperature) is used during the MD-DMS simulations. We apply ten cycles for each MD-DMS to measure the storage and loss shear moduli as well as damping factor δ, fitted by Equation (3).

Cluster and percolation analysis

We use the van Hove function $p(u, \varDelta t)$ to conduct statistics on atomic displacements. The van Hove function $p(u) \equiv [P(u + \varDelta u) - P(u)]/\varDelta u$ is used, in which $P(u)$ is the cumulative distribution quantifying the probability of finding the value $X \leq u$. We select one cycle of MD-DMS as the time interval $\varDelta t$. We define a displacement threshold u_c. In $P(u)$, if u is greater than u_c, it is considered a mobile atom.

We determine u_c by the displacement of the mean square displacement corresponding to the peak of Non-Gaussian Parameter. Based on Einstein's theory of Brownian motion, diffusion under equilibrium conditions results from the random walk of particles driven by thermal fluctuations. This process is characterized by two important properties. (i) Its mean squared displacement (MSD) increases linearly with time. The diffusion constant is correspondingly defined by the relation $D = \frac{\langle r^2(t) \rangle}{2dt}$, where *d* represents the dimensionality of the system. (ii) The probability distribution functions (PDFs) of position increments are Gaussian[4, 5]. However, in complex and disordered systems such as polymer networks, living cells, colloidal glasses, and active gels, diffusion often fails to satisfy the above two properties, manifesting as nonlinear MSD scaling and, importantly, significant non-Gaussian displacement distributions. Contrary to Gaussian behavior, non-Gaussianity indicates the presence of more complex dynamics associated with extreme events, critical phenomena, phase transitions, and other emergent behaviors. In such complex media, diffusive non-Gaussianity reflects structural or dynamic heterogeneity, serving as a key parameter for identifying anomalous mechanisms, describing rare events, and establishing statistical inferences. These deviations are quantitatively captured by the non-Gaussian parameter

$$\mathrm{NGP}(t) = \frac{3\langle (r(t)-r(0))^4 \rangle}{5\langle (r(t)-r(0))^2 \rangle^2} - 1 \qquad (4),$$

which serves as a key measure for quantifying dynamic heterogeneity[6, 7]. We take different temperatures for isothermal treatment and calculate NGP during the isothermal process, obtaining an approximate value of 0.9Å for u_c. The specific data is shown in Supplementary Fig. 2.

Then we define another important parameter, r_c, using the radial distribution function *g(r)*. We take the first minimum value of the radial distribution function as r_c. For percolation and cluster analysis[8, 9, 10], r_c=3.5Å (Supplementary Fig. 1a, the value for amorphous). Atoms with distances less than r_c are defined as belonging to the same cluster. Through this method, a series of clusters containing different atoms are identified. And the cluster with the largest number of atoms is defined as LC, and the cluster with the second most atoms is defined as SLC (Supplementary Fig. 3).

Based on u_c and r_c, we perform cluster analysis on the mobile atoms in the system and record the temperature-dependent evolution of LC and SLC cluster sizes to investigate the percolation phenomenon in the system. We apply the criterion to determine the percolation temperatures is that the size of LC is 100 times that of SLC.

Cluster alignment for crystalline cluster identification

To accurately identify crystalline clusters within mixed disordered and ordered GST structures, we employ a two-step cluster alignment strategy. We design this approach systematically to detect and classify crystalline regions based on well-defined structural templates and rigorous alignment criteria.

The first step, Template Extraction, involved analyzing highly crystallized GST configurations to extract representative local structures. Using the pair alignment mode[11, 12], individual clusters are compared against each other with a stringent alignment score threshold set at 0.06. This low value ensures that only configurations with a high degree of similarity are grouped together. From these aligned clusters, we select the most frequently occurring motifs as reference templates. These templates effectively capture the dominant atomic arrangements within the crystalline phases and provide a reliable basis for subsequent identification in disordered systems.

In the second step, Crystalline Cluster Identification, we apply the pre-defined templates to mixed GST structures using the template alignment mode. A slightly higher alignment score cutoff of 0.08 is adopted to accommodate structural variations in less ordered environments. Clusters with alignment scores below this threshold were classified as crystalline. This score is located around the first low-score peak of the score distributions of Ge, Sb, and Te (see Fig. S26), avoiding arbitrariness of choosing score. To maintain structural realism, we permit each identified cluster to contain no more than two vacancies, preserving the essential geometry of the crystalline phase while allowing for minor defects.

This two-step procedure enables robust and precise discrimination of crystalline motifs within heterogeneous environments, forming a foundation for quantitative analysis of crystallization processes in phase-change materials such as GST.

1.2 Experiments: Laser induced crystallization under ultrasonic vibrations

The experimental set-up is shown in Supplementary Fig. 20. Ultrasonic excitation was generated with a commercial quartz crystal microbalance (QCM) instrument (openQCM Q-1, Novaetech, Italy) with a fundamental 5 MHz AT-cut quartz sensor (Novaetech). The instrument passively interrogates the crystal by sweeping around the resonance frequency of the *quartz* sensor. The actuation uses an AD9851 DDS/DAC frequency synthesizer for sinusoidal waves; detection uses an AD8302 gain/phase detector for measuring the resonance frequency and quality factor Q (the bandwidth at -3dB), as well as the dissipation $D = 1/Q$ (Ref[13]). The quartz sensor has a gold-electrode active area ~6 mm in diameter (corresponding to an area of $A \approx 2.8 \times 10^{-5} m^2$). Measurements were performed in air on the sensor with the sample thin-film deposited on its upper electrode. In the deposited thin films, the $Ge_2Sb_2Te_5$ layer with the thickness of 100 nm is sandwiched by a 10 nm thermal barrier as well as a 100 nm capping layer of $(ZnS)_{80}(SiO2)_{20}$, to prevent the heat loss and potential evaporation, respectively. The accessible frequencies are the fundamental frequency 5 MHz of the quartz and its overtone (n= 3, 5, 7) frequencies 15, 25, and 35 MHz. Such a device allows us to apply laser pulses to induce crystallization of amorphous thin films under the ultrasonic vibrations and in-situ monitor the crystallization kinetics by measuring the changes in reflectivity of the sample. Simultaneously, the frequency and dissipation of the ultrasonic waves are monitored to ensure a stable frequency during the testing period, as shown below:

Supplementary Table 1. Frequencies and dissipation during the measurements

Set frequencies	5 MHz	15 MHz	25 MHz	35 MHz
Measured frequency (standard deviation) (MHz)	4.994674 (4×10^{-6})	14.973056 (8×10^{-6})	24.951332 (2×10^{-6})	34.928438 (9×10^{-6})
Dissipation *D* (standard deviation) ($\times 10^{-5}$)	9.83 (0.01)	3.047 (0.006)	1.927 (0.005)	4.020 (0.002)

In the hardware, the AD9851 has a DAC full-scale current of nominal 10 mA with voltage-compliance of -0.5 to 1.5 V (Ref.[14]). The current-to-voltage resistor is 100 Ohm at IOUT pins, producing a voltage ~ 1 V_{p-p} (~ 0.35 V_{rms}) for exciting the QCM sensor, which is within the linear operating regime. The quartz sensor can be modeled by a Butterworth-van Dyke (BvD) circuit, consisting of the RLC series (R_1, L_1, C_1) branch in parallel with C_0. The electrical equivalent form related to the quality factor Q and dissipation D reads: $Q = 1/D = \omega_0 L_1/R_1$, where $\omega_0 = 2\pi f_0$ and $L_1 = 1/(w_0{}^2 C_1)$ (Ref.[15]). Using f_0 = 5 MHz, and D = 9.83E-5, and assuming a typical range of C_1 = 0.02 to 0.03 pF, one can estimate R_1 ~ 125 Ohm. Then the voltage on the quartz is $V_{quartz} = 0.35 \times R_1/(R_1 + R_s) = 0.25$ V, where R_s is an impedance, assumed 50 Ohm, in series with the quartz. The power driving the vibration in the motional branch is estimated as $P = V_{quartz}{}^2/R_1 \sim 0.5$ mW, which is a sub-mW power. The device does not record the amplitude of the displacement of the ultrasonic vibrations. For estimating the amplitude, we use the time average power flux over one cycle $P = \frac{1}{2} A \cdot \Re\{Z_L\} \cdot v_0{}^2$, where $A = 2.8 \times 10^{-5} m^2$ is the gold-electrode active area, the surface particle velocity $v_0 = \omega_0 u_0$ is the product of angular frequency ω_0 and the surface displacement amplitude u_0, and $\Re\{Z_L\} \approx (\pi Z_q/2) \cdot D$ is the real part of the load mechanical impedance Z_L per unit area under the small-load approximation[16], which is related to the dissipation D, and the standard quartz acoustic impedance $Z_q = 8.8E6\ kg \cdot m^{-2} s^{-1}$. Rewriting these equations gives $u_0 = \frac{1}{2\pi f_0} \sqrt{\frac{4P}{A\pi Z_q D}} \approx 5\ nm$. The estimated displacment corresponds to a motion on the order of magnitude of nanometer-scale, which is consistent with a finite element simulation in the literature[17].

1.2.1 Analysis and discussion of possible non-resonant macroscopic mechanisms

Macroscopic strain and overtone analysis**:** To rule out strain-induced crystallization or non-resonant frequency-dependent acoustic coupling, we quantitatively analyzed the acoustic energy delivered across the overtones. Based on our measured QCM data logs, the dissipation factor D_n drops significantly at higher overtones *n* (from roughly $D_1 = 9.833 \times 10^{-5}$ at 5 MHz (*n* =1), to $D_3 = 3.0455 \times 10^{-5}$ at 15 MHz (n=3), to $D_5 = 1.9271 \times 10^{-5}$ at 25 MHz (*n* =5)). Note that D_n drops at the nearly the same rate as the drop of ωC_n, where ω is the frequency and C_n is the motional capacitance that drops with *n* as $C_n = C_1/n^2$ (Cerda, *Understanding Quartz Crystals*

and Oscillators (Artech House, 2014)). Thus, the motional resistance $R_n = D_n/\omega C_n$ can be rewritten as $R_n = nD_n/\omega_1 C_1$ (where ω_1 and C_1 are constant). Since nD_n remains stable (only ~2% between n = 1 and 5), the motional resistance remains nearly constant at these frequencies. As a result, the delivered acoustic power is nearly identical at these frequencies. As the mechanical strain $\varepsilon = \frac{v_0}{v_{sound}} = \frac{1}{v_{sound}}\sqrt{\frac{4P}{A\pi Z_q D}}$ is proportional to $1/\sqrt{D}$ at the constant power P, the acoustic strain exerted on the film at 25 MHz is indeed more than two times higher than the strain at 5 MHz.

If the accelerated crystallization were an artifact of macroscopic strain or acoustic coupling, the 25 MHz excitation would have induced the largest crystallization acceleration. Instead, the strongest enhancement occurred at 5 MHz, which corresponds to the lowest macroscopic strain, but aligning with the β-relaxation regime extrapolated using the activation energy from our simulations. This inverse relationship between the macroscopic strain and the observed acceleration rules out the frequency-dependent acoustic strain or coupling as the physical origin.

Control of substrate and thermal transport**:** All frequencies (0 to 35 MHz) were tested on the exact same deposited film stack, which included a 10 nm thermal barrier and a 100 nm capping layer to prevent heat loss and evaporation. Since the active sensor area with 6 mm in diameter is much larger than the microscopic laser-irradiated spots (~1 μm spot size with a 5 μm pitch), measurements at different frequencies were performed at well-separated locations (> ~1 mm distance) on the identical film. This spatiotemporal multiplexing effectively zeros out sample-to-sample variations in interfacial thermal resistance, substrate properties, and film thickness.

To prove that the experimental conditions prevented "substrate effects", "additional heating", or variations in "thermal transport", we have analyzed and compared the two independent PTE measurements at 5 MHz using a coarse and a fine temporal- and power-resolution (see Fig. S23). Coarse-step PTE measurements used a 30×10 parameter array, spanning pulse widths of 10 to 10^5 ns and powers of 0 to 20 mW; fine-step measurements employed a denser 40×20 array over a narrowed power range of 14 to 20 mW. Despite a higher density of laser pulses required for the fine-step measurement, the contrast curve superimposes excellently with the coarse-step measurement. If there were cumulative heating, the baseline temperature of the film would increase more rapidly during the fine scan, leading to an earlier

onset of crystallization. The precise alignment of the onset of crystallization confirms that thermal transport out of the film is highly efficient, preventing any cumulative laser-induced heating. Furthermore, the reproducibility of these independent measurements confirms that the film stack and QCM substrate are highly uniform, ruling out spatial "substrate effects". Therefore, the observed acceleration at 5 MHz is not from spatial heterogeneities of the film, substrate, or measurement induced thermal artifacts.

Acoustic heating**:** The time-averaged acoustic power driving the vibration is estimated at the order of 0.5 mW (SI-Sec.1.2). This sub-milliwatt power is distributed across the entire macroscopic active area, making the acoustic energy density vanishingly small compared to the thermal energy deposited by the highly focused 14.2 to 18.4 mW nanosecond pump laser. Global acoustic heating is therefore negligible. If there were acoustic heating or altered thermal transport at 5 MHz, the baseline temperature of the film would rise, shifting the onset of crystallization to shorter laser pulse widths. However, the temporal onsets for 0 MHz and 5 MHz are virtually identical (Fig. 4c and Fig. S22), unambiguously ruling out the global acoustic heating artifact. Since the onset is the same, the 5 MHz acceleration occurs only in the growth phase (shifting the contrast from 42% up to 47% faster than 0 MHz). This is well consistent with the MD simulations, where the ultrasonically modulated mobility via percolation substantially enhanced the growth (Fig. 3c).

1.3. Discussion of frequency mismatch between simulations and experiments

The large difference in frequency likely reflects the vastly different time and length scales accessible to MD simulations and experiments. MD simulations access local atomic dynamics on nanosecond timescales limited by computational power, whereas experiments probe macroscopic relaxations over much larger length and timescales. Therefore, the mismatch in absolute frequencies is not unexpected. However, their temperature dependence, i.e., the activation energy, should reflect the intrinsic material properties in experiments. To examine this point further, we performed additional MD simulations at frequencies of 3.3 GHz (300 ps) and 10 GHz (100 ps). The results are shown in Fig. S24. For each frequency, the β-relaxation

temperature was identified from the mobile-particle percolation, defined by the criterion $LC_{mobile}/SLC_{mobile} = 100$.

As shown in Fig. S25, the temperature dependence of frequencies yields an activation energy of 118 ± 3 kJ/mol. With this activation energy, we now turn to the experimental DMS measurements of GST (Peng et al., *Sci. Adv.* 6, eaay6726 (2020)), where the β-relaxation temperature measured at 1 Hz is approximately 420 K (Fig.2 of Ref. Peng et al., *Sci. Adv.* 6, eaay6726 (2020)). By extrapolating the relaxation frequency by about six and a half orders of magnitude, from 1 Hz at 420 K of experimental DMS measurements to 5 MHz (assuming a simple Arrhenius behavior), we estimate a corresponding temperature increase of approximately 351 K, pointing to a temperature of around 771 K. As shown in Fig. R2, this temperature lies near the middle of the supercooled liquid region of GST, well above $T_g \approx 473$ K (Pries et al. Adv. Mater. 31, 1900784 (2019)) and below the melting temperature $T_m = 893$ K (Kalb et al. J. Appl. Phys. 93, 2389–2393 (2003)). This is a reasonable temperature range for crystallization during laser-heating experiments. Although this is only an order-of-magnitude estimate, it suggests that a MHz β-relaxation in the supercooled liquid of GST is physically plausible.

2. Supplementary figures

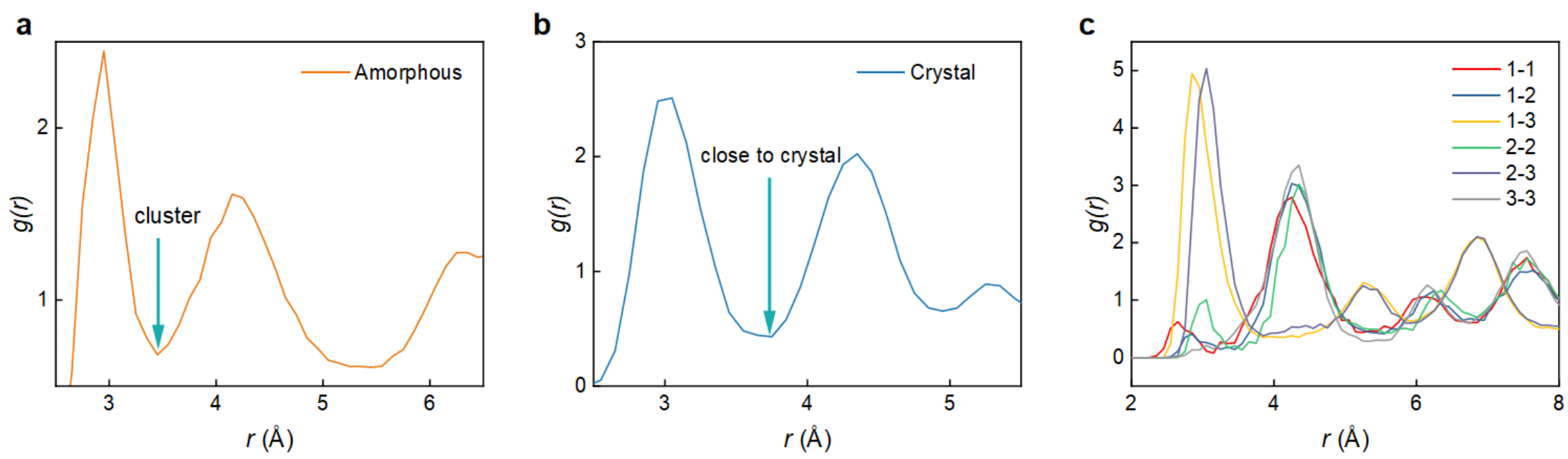

Supplementary Fig. 1. The radial distribution function *g(r)* of GST. a. For the amorphous GST, the r_c is 3.5 Å, when the distance between atoms is below this value, atoms by definition belong to the same cluster. **b.** For the crystal GST, the r_c is 3.8 Å, when the distance between atoms is below this value, atoms are considered to be close neighbors of the crystal. **c**. The

effect of different atomic pairs on the radial distribution function. The numbers 1, 2 and 3 respectively represent Ge, Sb and Te.

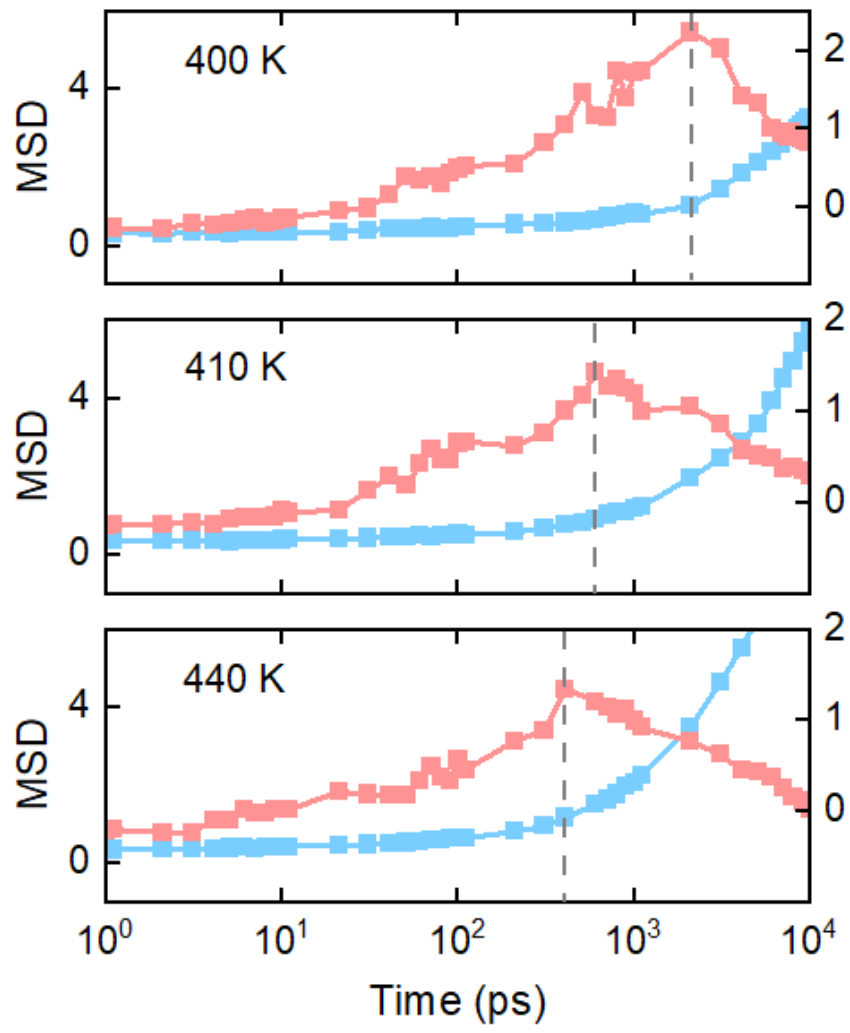

Supplementary Fig. 2. Value of u_c. The u_c is determined by the displacement u of atoms corresponding to the peak of Non-Gaussian Processes (NGP) (dashed line). Blue squares represent mean square displacement (MSD). Red squares indicate NGP, characterizing the inhomogeneity of dynamics. Mobile atoms have displacement greater than u_c.

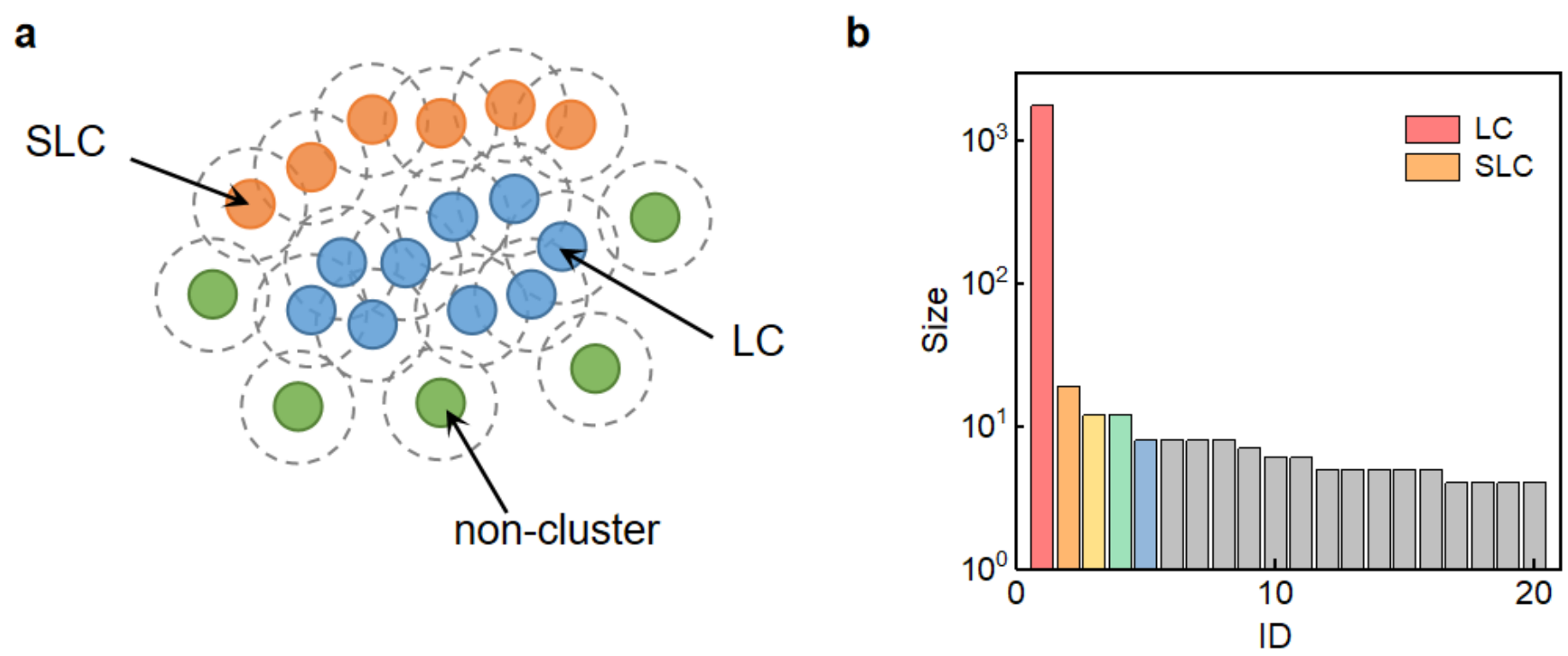

Supplementary Fig. 3. Cluster analysis. a. The process of cluster analysis for the largest (LC) and the second largest (SLC). Dashed line indicates the connectivity criterion r_c. **b**. The data of the top 20 clusters are taken according to the cluster size.

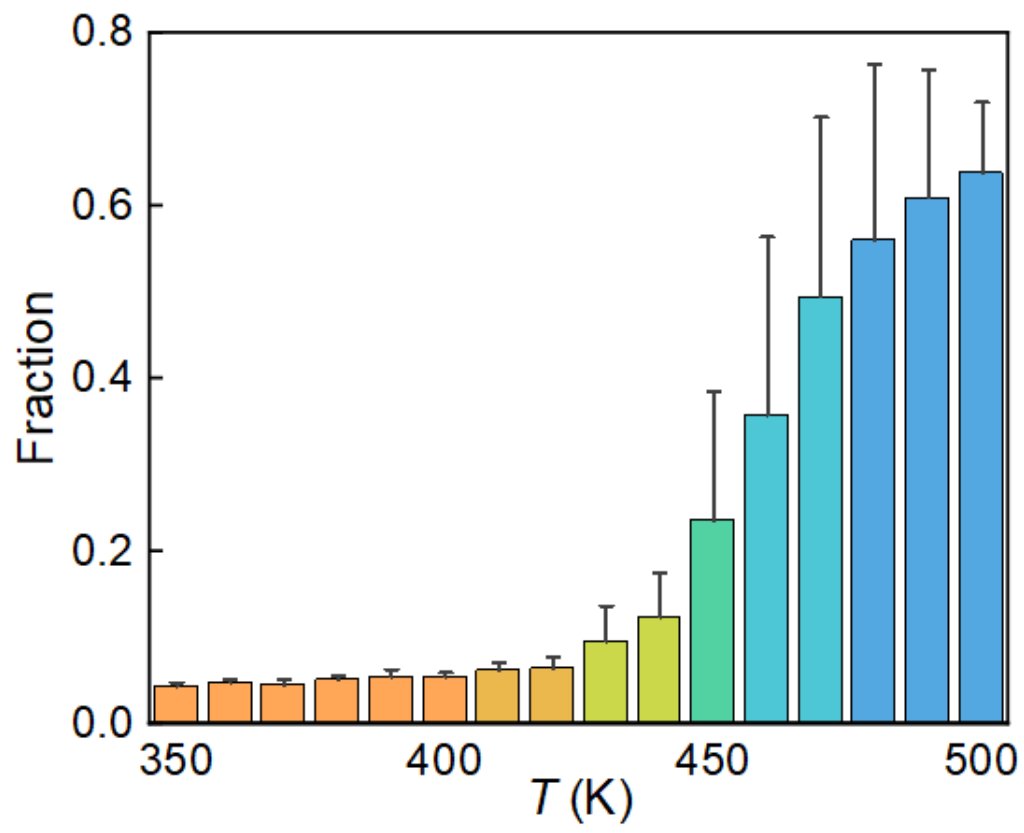

Supplementary Fig. 4. Crystallized atoms fraction. Crystal fraction with error statistics during MD-DMS.

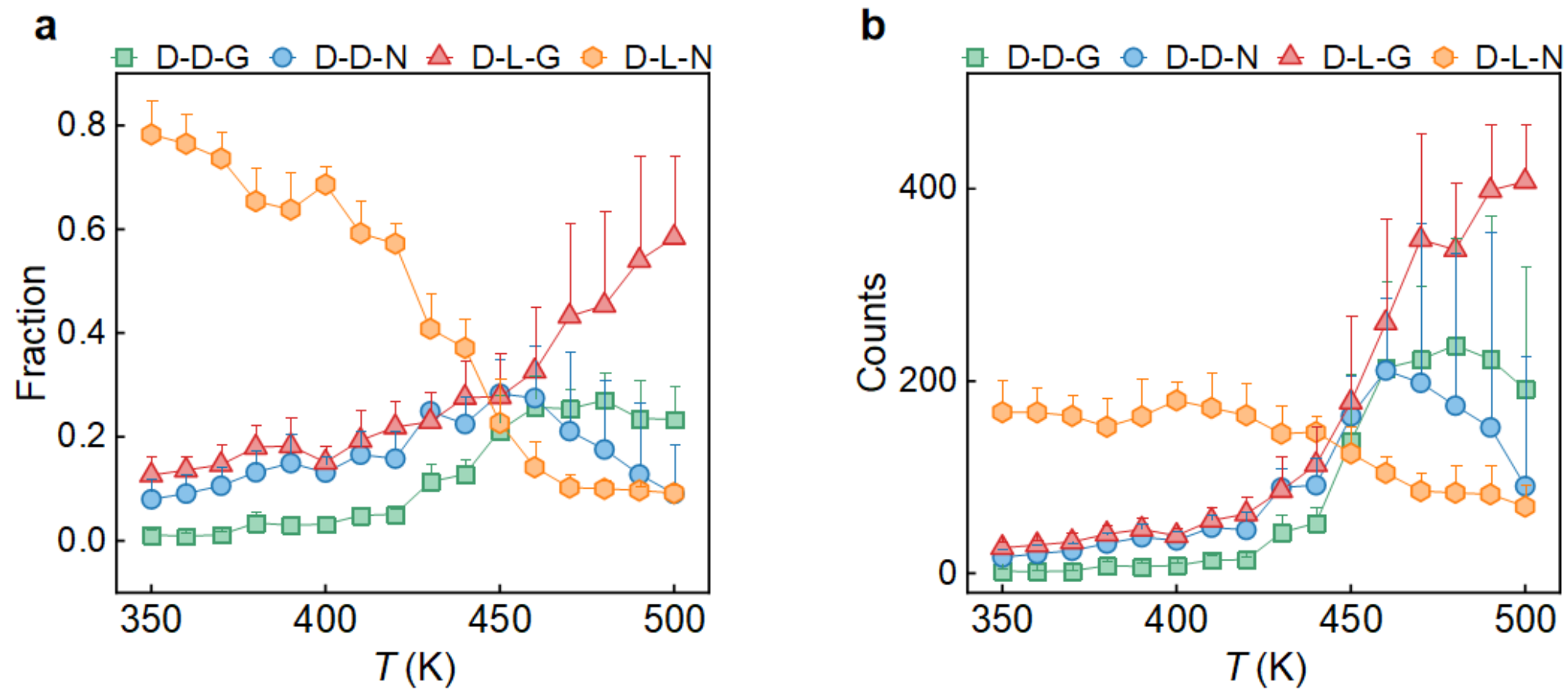

Supplementary Fig. 5. Analysis of different crystal types during DMS at each temperature. **a.** New crystalline atom percent with error statistics of distinct crystallization types during DMS at each temperature. **b**. New crystalline atom counts with error statistics of distinct crystallization types during DMS at each temperature.

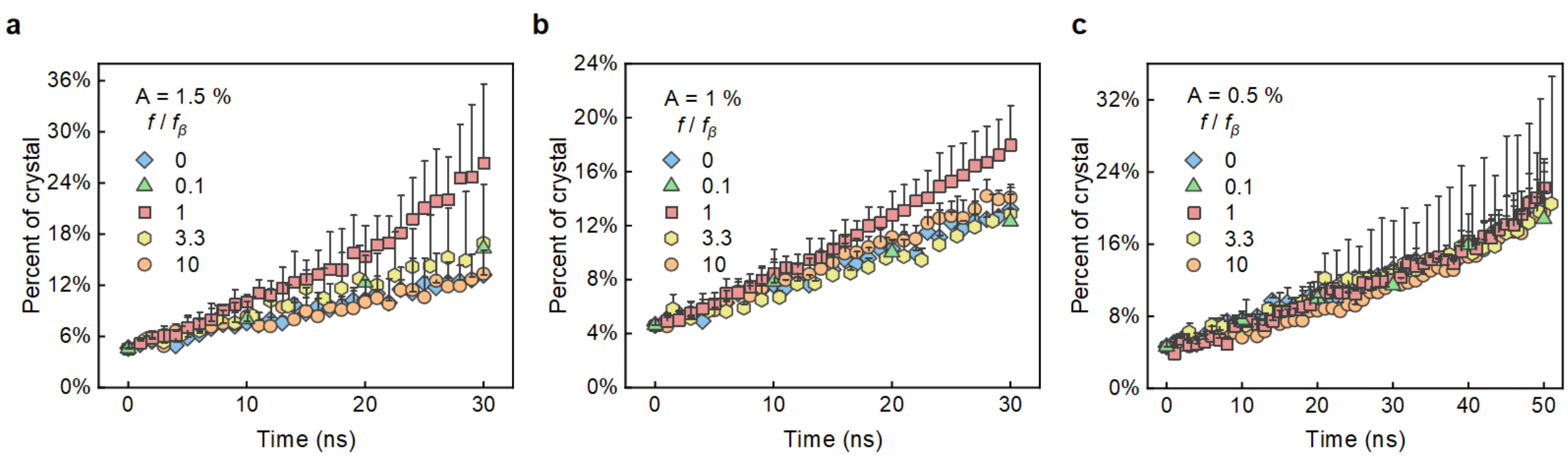

Supplementary Fig. 6. The proportion of crystals after ultrasonic vibration treatment at different amplitudes. Crystalline percent with error statistics versus time at ultrasonic amplitudes of 1.5%, 1% and 0.5%.

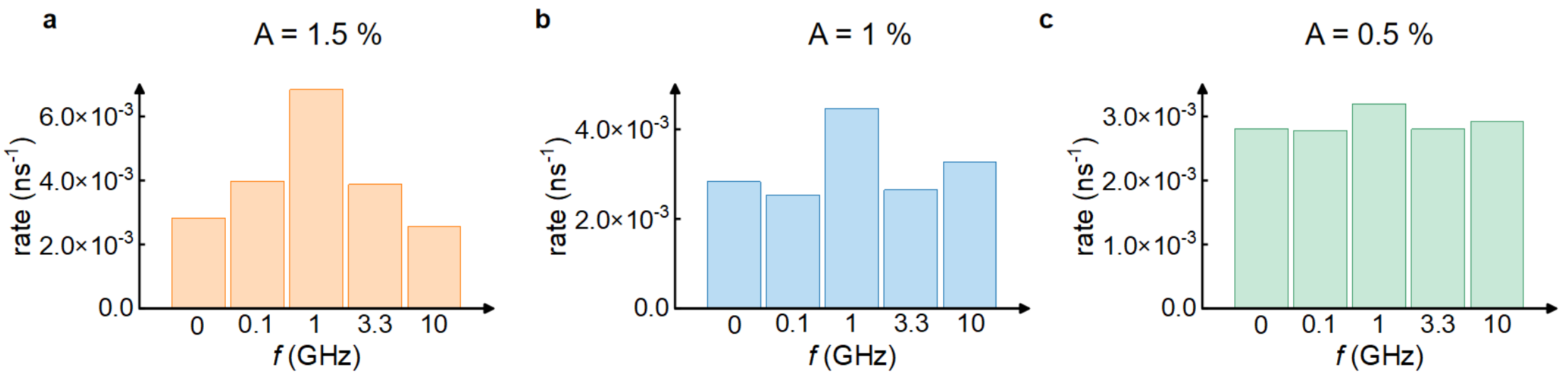

Supplementary Fig. 7. The crystallization rate after ultrasonic vibration treatment at different amplitudes. The crystallization rate of different frequencies at different amplitudes shows that the crystallization rate corresponding to the 1GHz frequency is the largest, regardless of which amplitude.

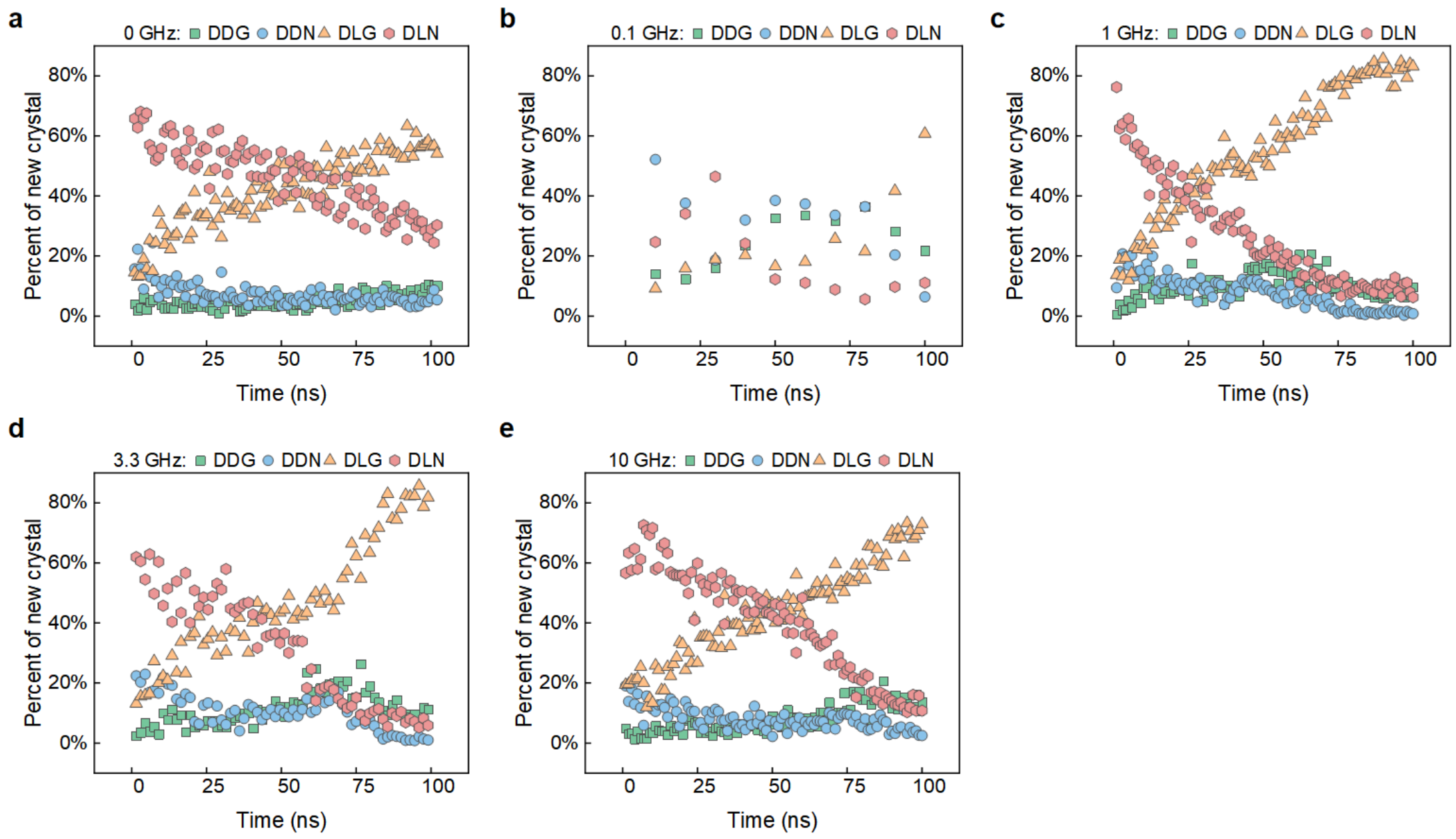

Supplementary Fig. 8. Analysis of four crystal classifications with an ultrasonic vibration amplitude of 1%. The percent of the three crystal types in 100ns changes with time at different frequencies. From **a** and **c**, it can be seen that compared with the result without applying ultrasonic vibration, the proportion of diffusion-less nucleation crystallization at a frequency of 1GHz decreased significantly from the very beginning, while the proportion of diffusion-driven crystallization increased. This result is not obvious at other frequencies.(DDG means diffusion-driven growth, DDN means diffusion-driven nucleation, DLG means diffusion-less growth and DLN means diffusion-less nucleation.)

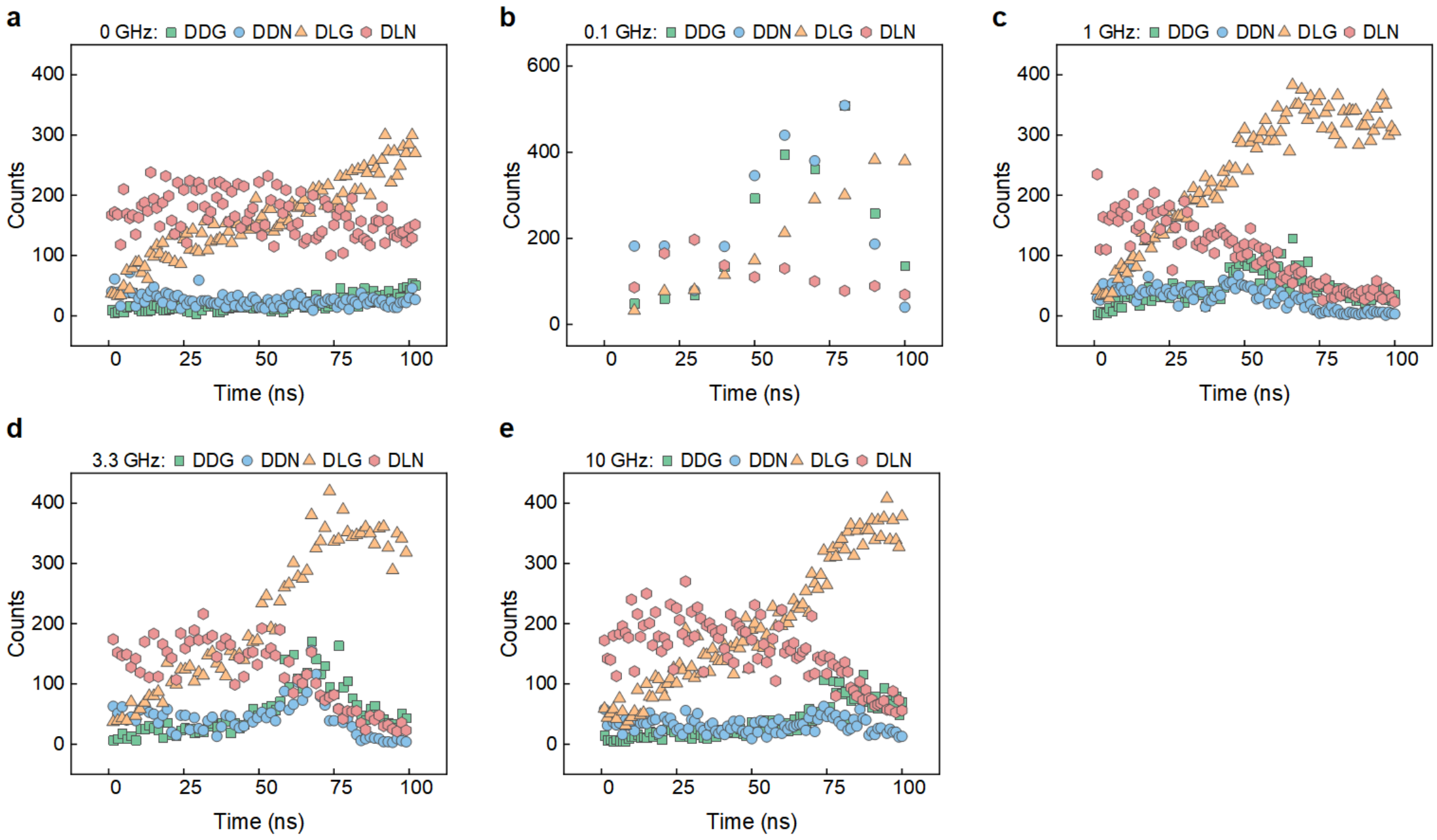

Supplementary Fig. 9. Analysis of four crystal classifications with an ultrasonic vibration amplitude of 1%. The counts of the four crystal types in 100ns changes with time at different frequencies.The result is consistent with Supplementary FIG. 6.

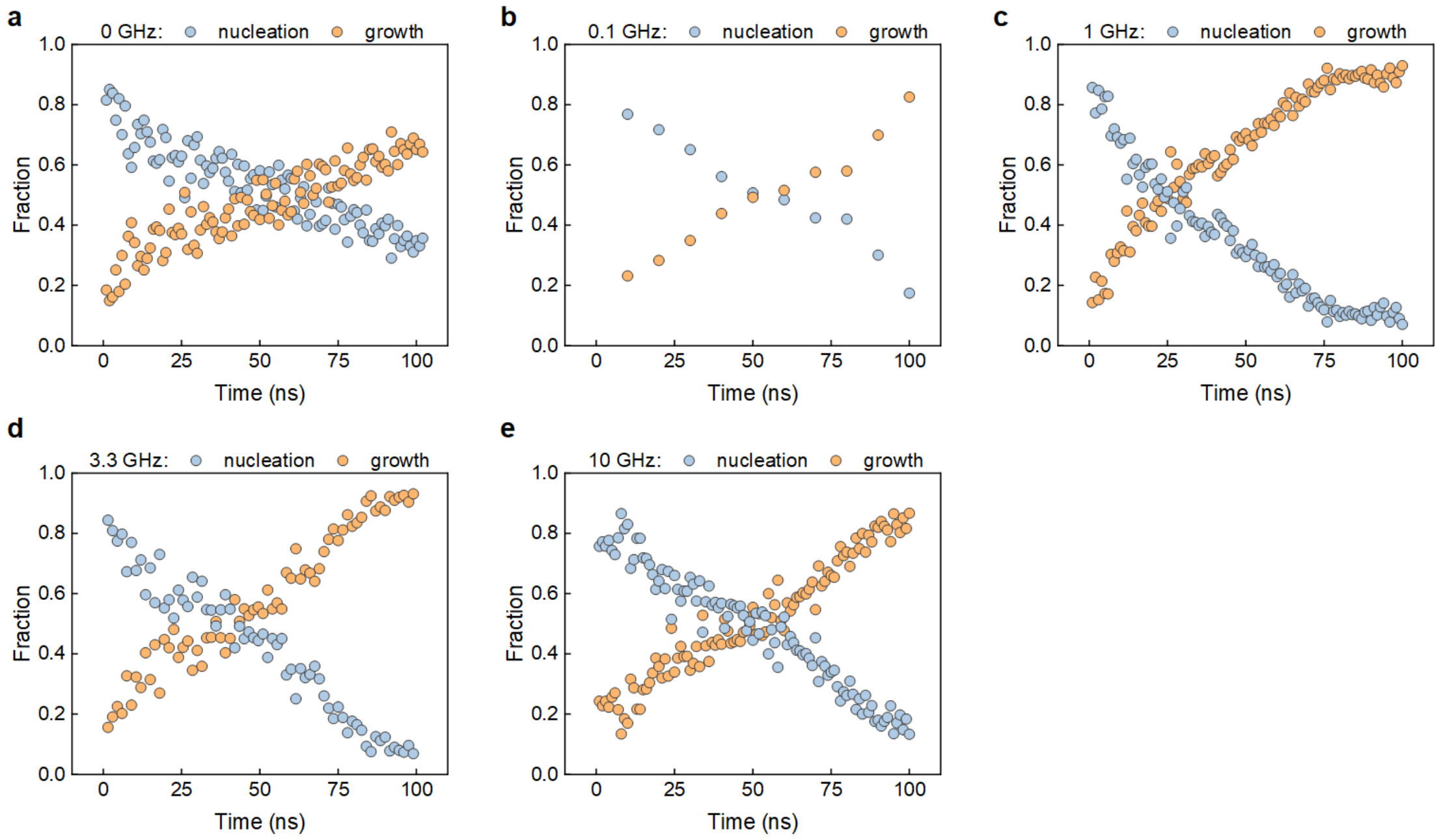

Supplementary Fig. 10. Analysis of nucleation predominant crystallization and growth predominant crystallization with an ultrasonic vibration amplitude of 1%. The percent of nucleation predominant crystallization and growth predominant crystallization in 100ns changes with time at different frequencies. It is worth noting that under the ultrasonic vibration of 1GHz, the transformation from nucleation-dominated crystals to growth-dominated crystals is significantly accelerated.

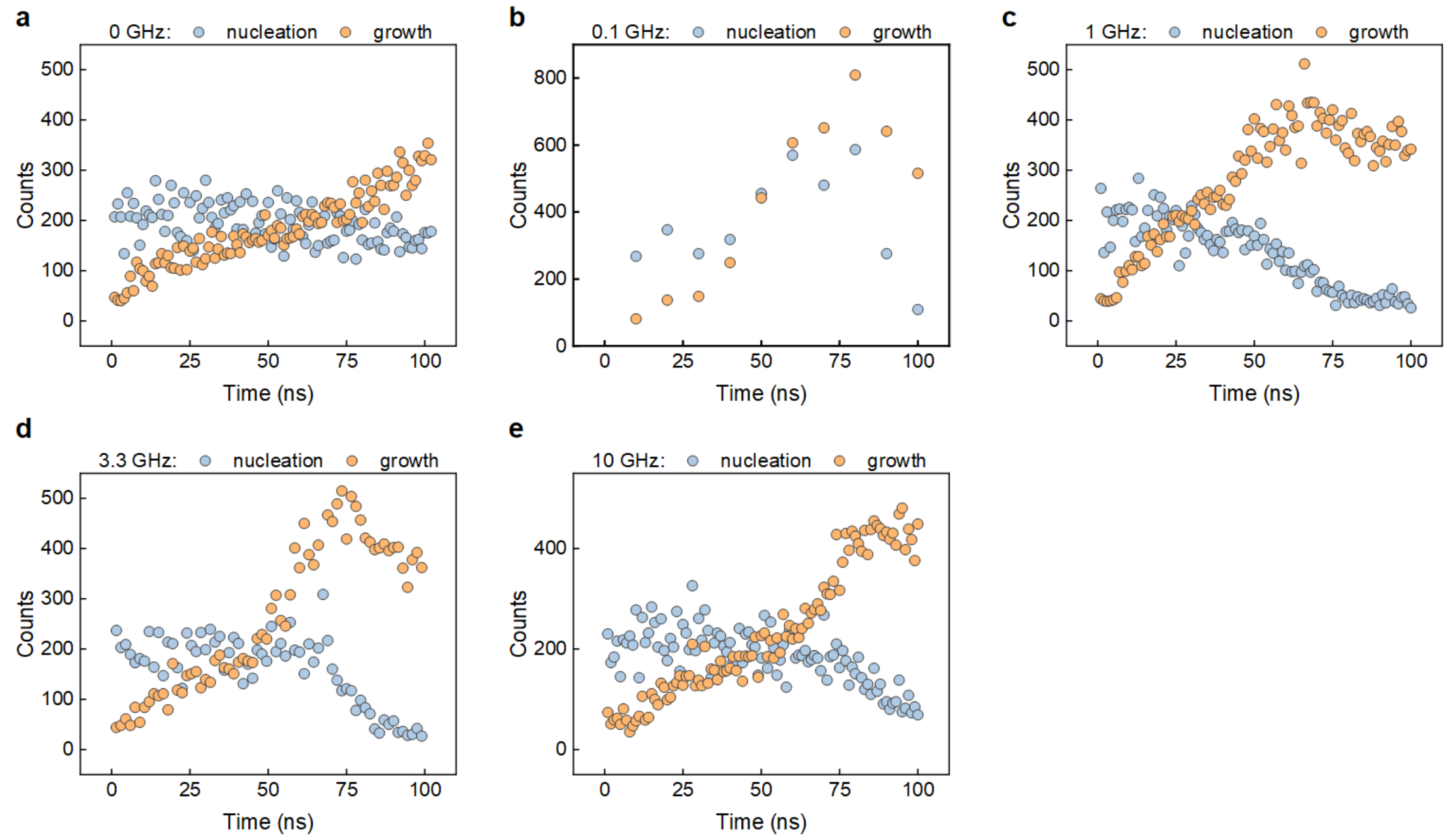

Supplementary Fig. 11. Analysis of nucleation predominant crystals and growth predominant crystals with an ultrasonic vibration amplitude of 1%. The counts of nucleation predominant crystallization and growth predominant crystallization in 100ns changes with time at different frequencies.

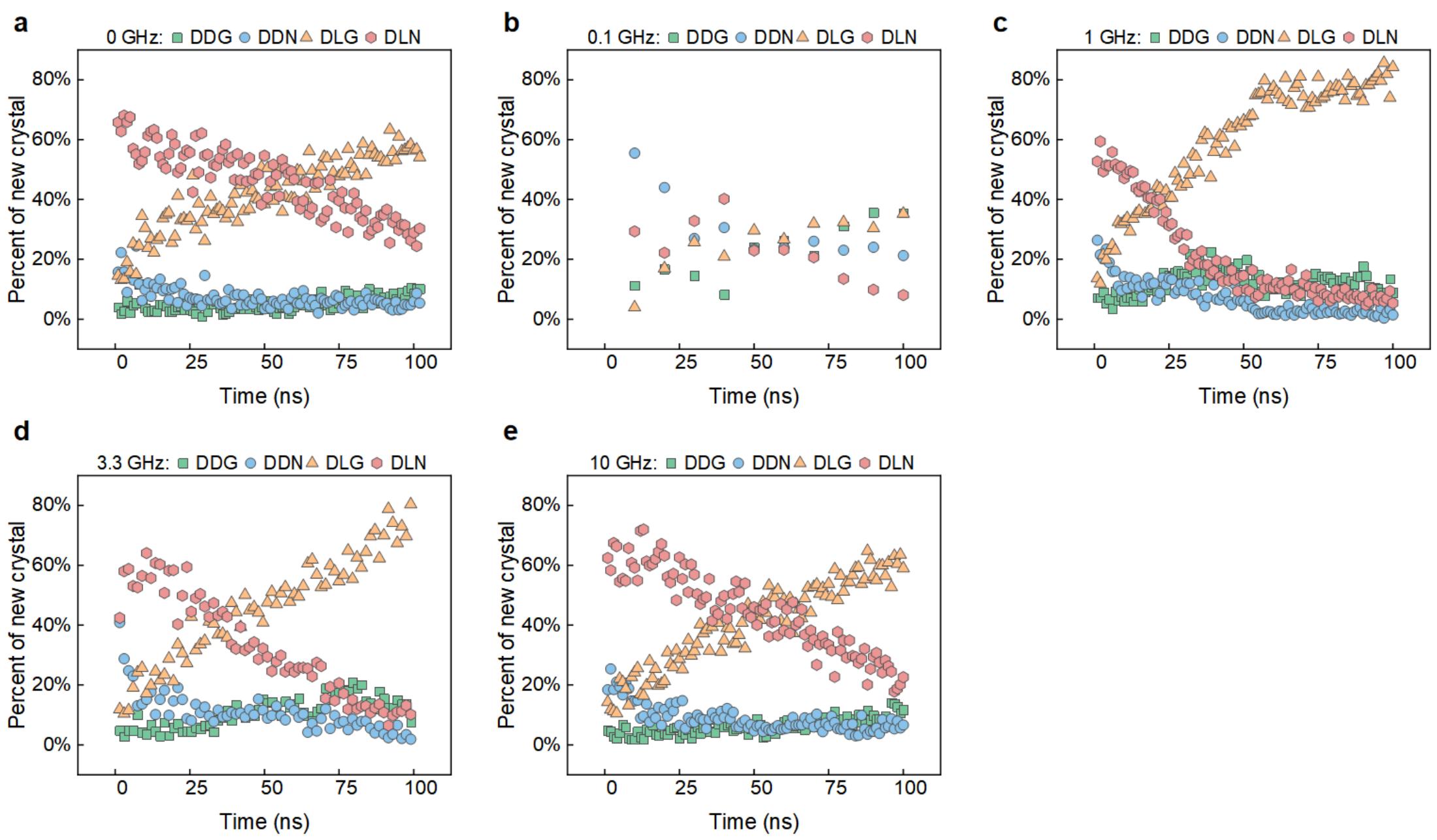

Supplementary Fig. 12. Analysis of four crystal classifications with an ultrasonic vibration amplitude of 1.5%. The percent of the four crystal types in 100ns changes with time at different frequencies. The result with an amplitude of 1.5% was consistent with the result with an amplitude of 1%, but the change at amplitude 1.5% was more dramatic.

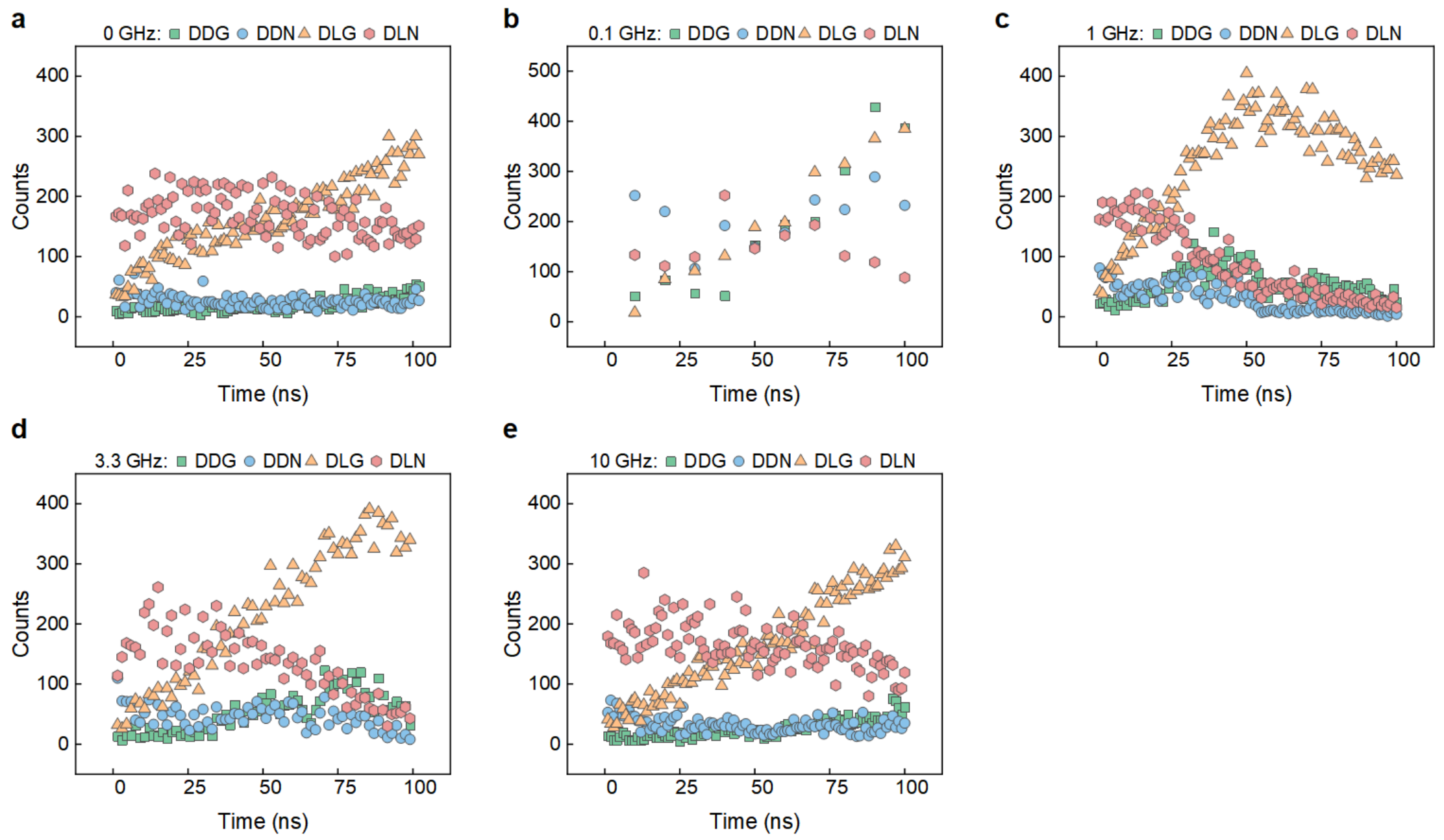

Supplementary Fig. 13. Analysis of four crystal classifications with an ultrasonic vibration amplitude of 1.5%. The counts of the three crystal types in 100ns changes with time at different frequencies.

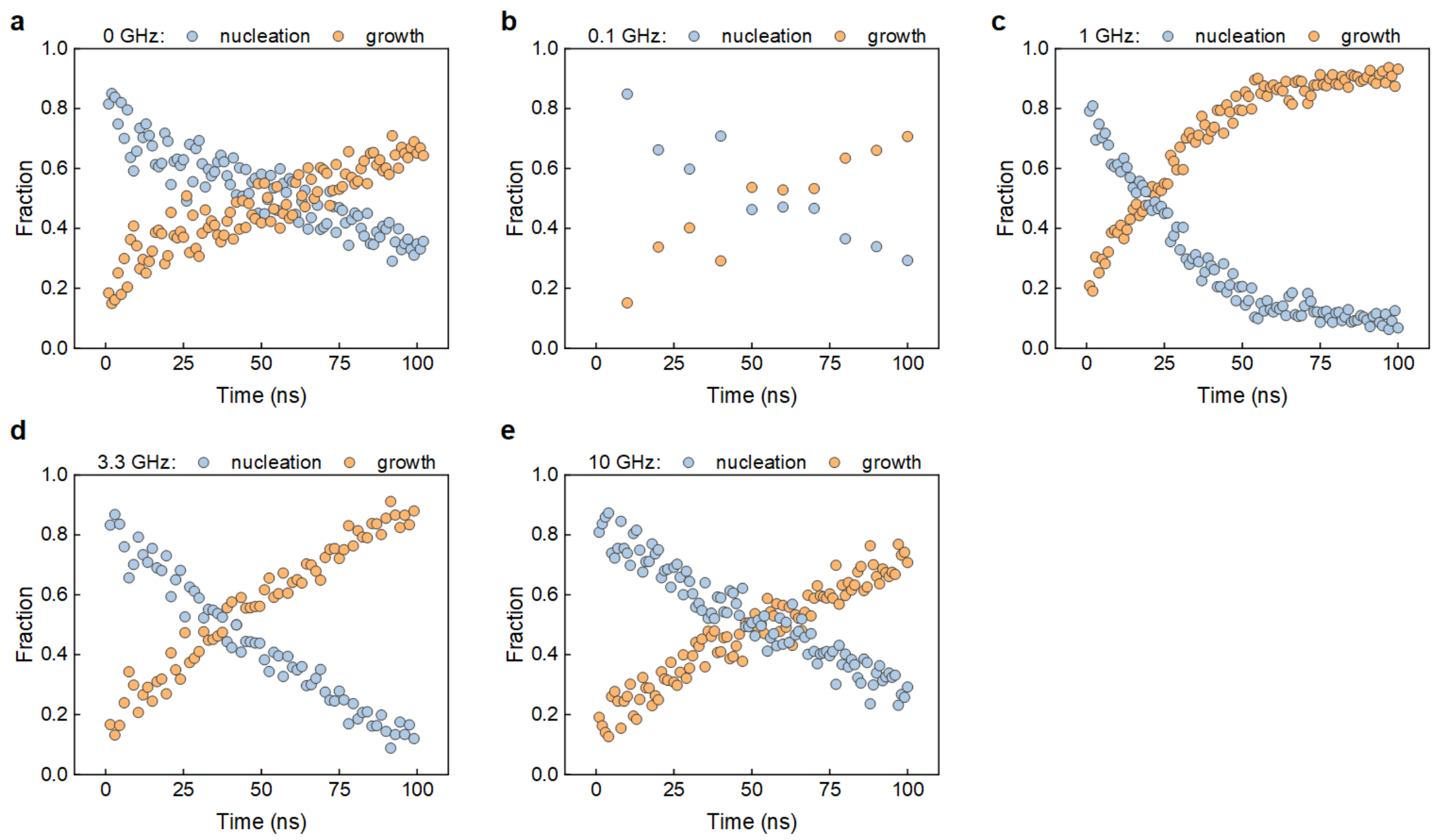

Supplementary Fig. 14. Analysis of nucleation predominant crystals and growth predominant crystals with an ultrasonic vibration amplitude of 1.5%. The percent of nucleation predominant crystallization and growth predominant crystallization in 100ns changes with time at different frequencies.

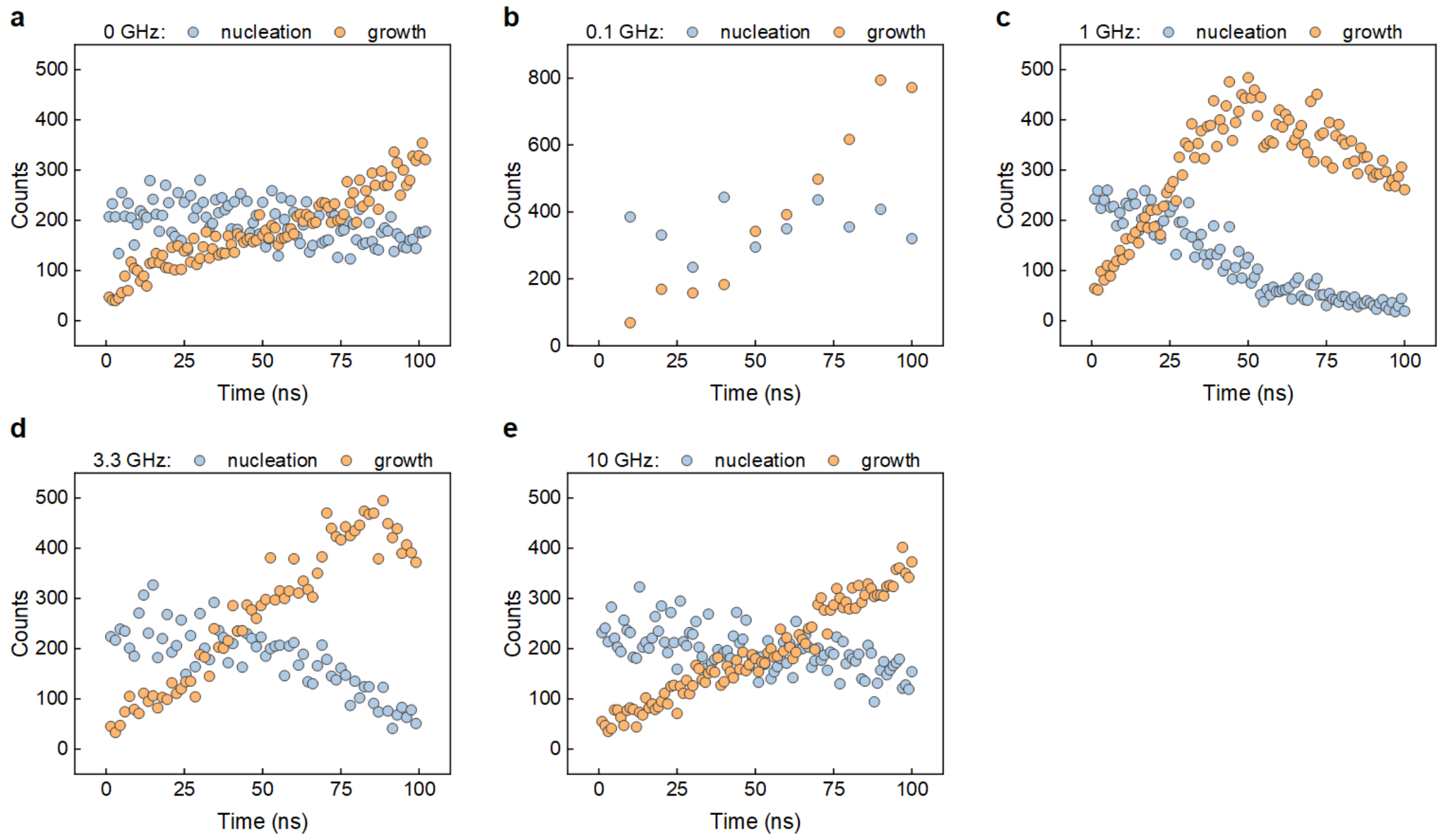

Supplementary Fig. 15. Analysis of nucleation predominant crystals and growth predominant crystals with an ultrasonic vibration amplitude of 1.5%. The counts of nucleation predominant crystallization and growth predominant crystallization in 100ns changes with time at different frequencies.

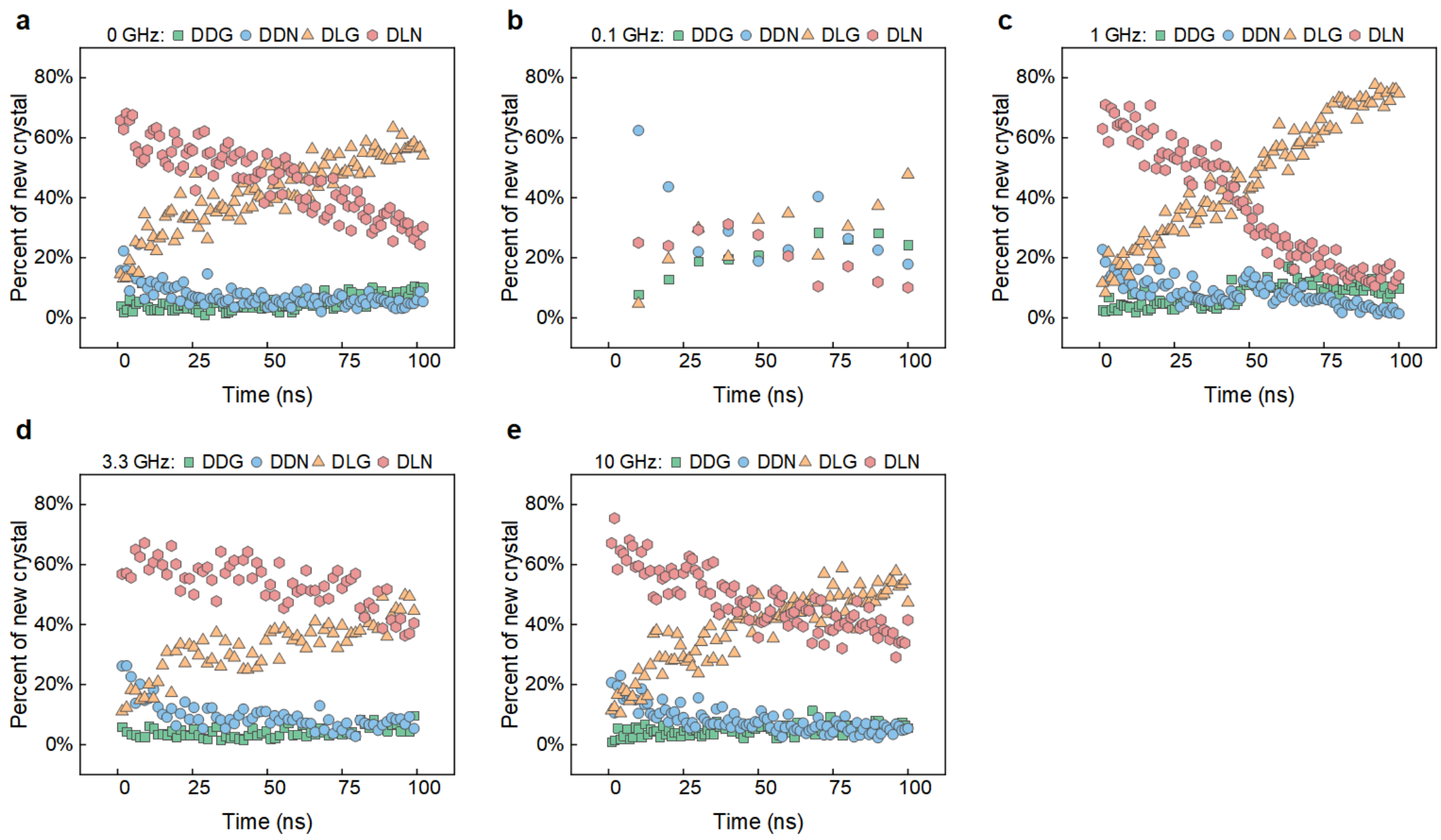

Supplementary Fig. 16. Analysis of four crystal classifications with an ultrasonic vibration amplitude of 0.5%. The percent of the four crystal types in 100ns changes with time at different frequencies. The result with an amplitude of 0.5% was consistent with the result with an amplitude of 1%, but the change at amplitude 0.5% was less dramatic.

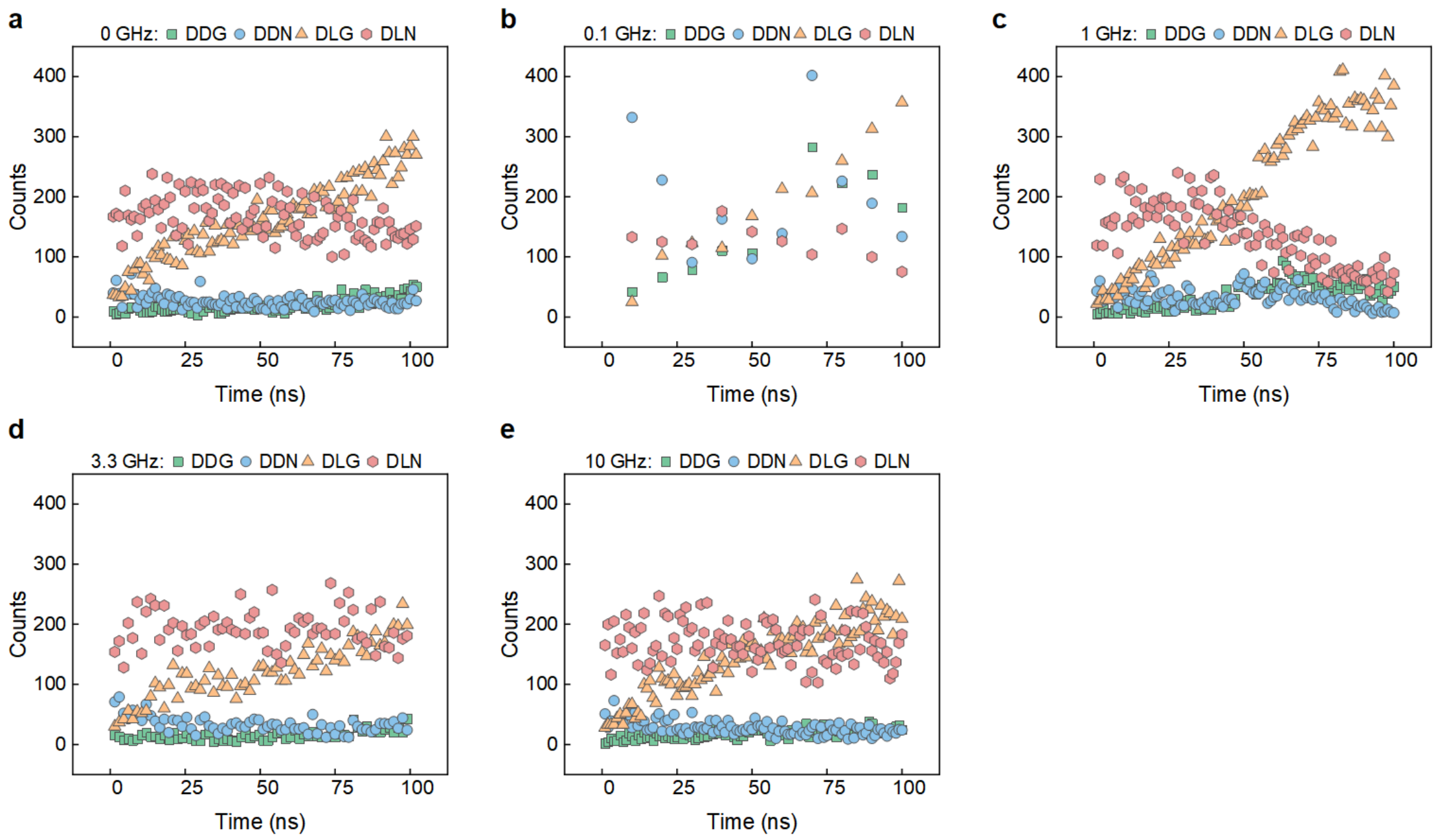

Supplementary Fig. 17. Analysis of four crystal classifications with an ultrasonic vibration amplitude of 0.5%. The counts of the four crystal types in 100ns changes with time at different frequencies.

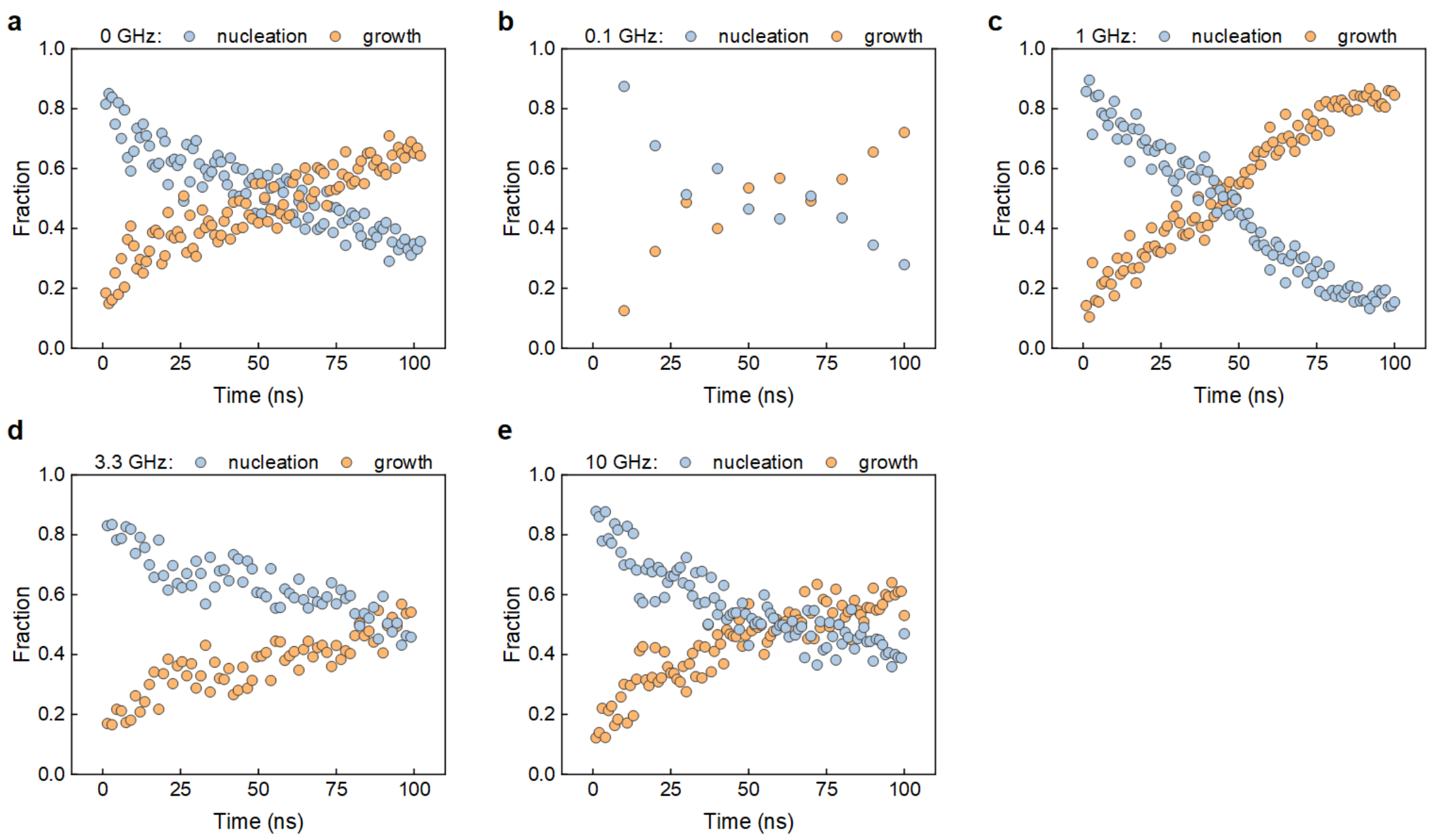

Supplementary Fig. 18. Analysis of nucleation predominant crystals and growth predominant crystals with an ultrasonic vibration amplitude of 0.5%. The percent of nucleation predominant crystallization and growth predominant crystallization in 100ns changes with time at different frequencies.

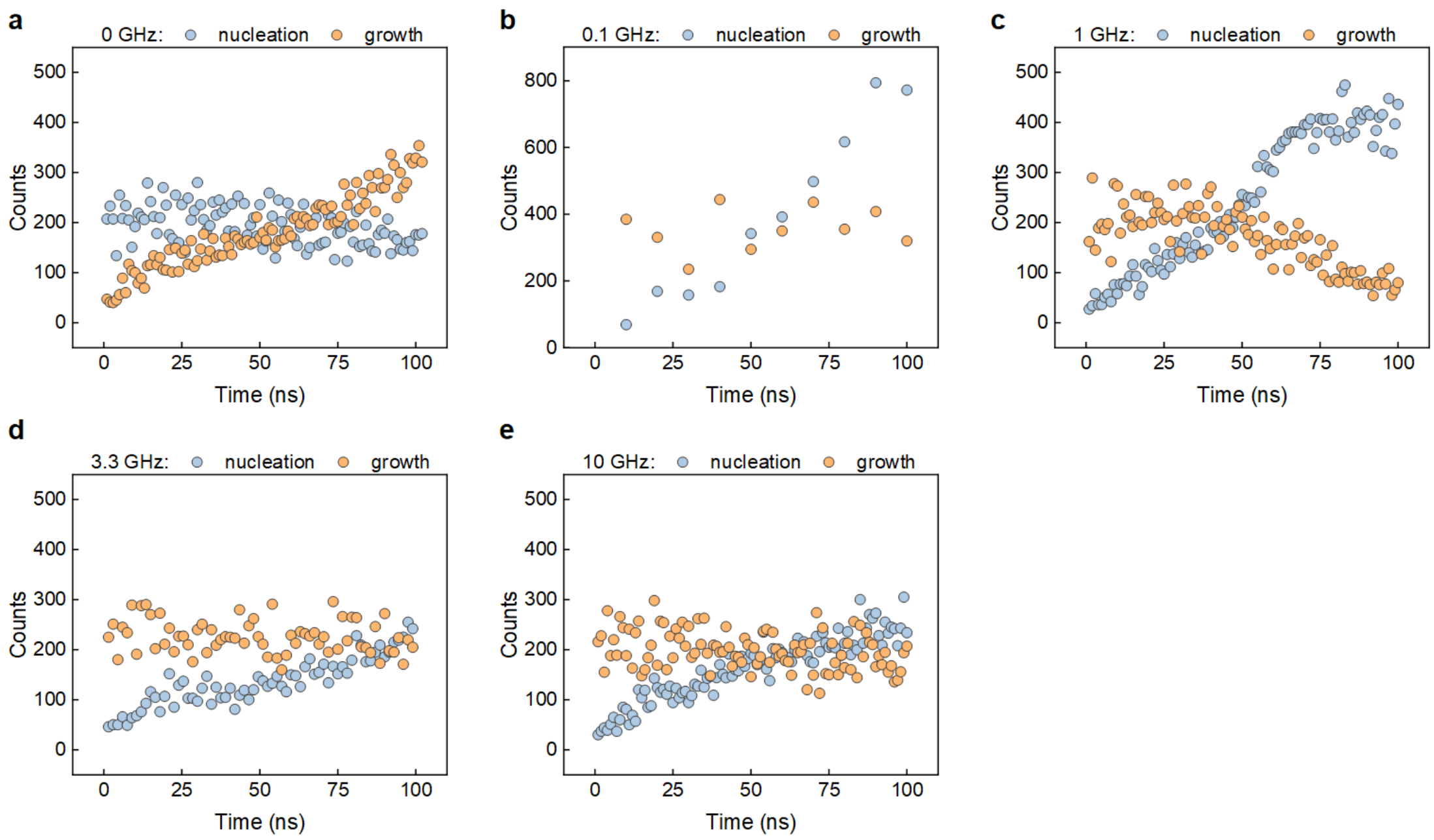

Supplementary Fig. 19. Analysis of nucleation predominant crystals and growth predominant crystals with an ultrasonic vibration amplitude of 0.5%. The counts of nucleation predominant crystallization and growth predominant crystallization in 100ns changes with time at different frequencies.

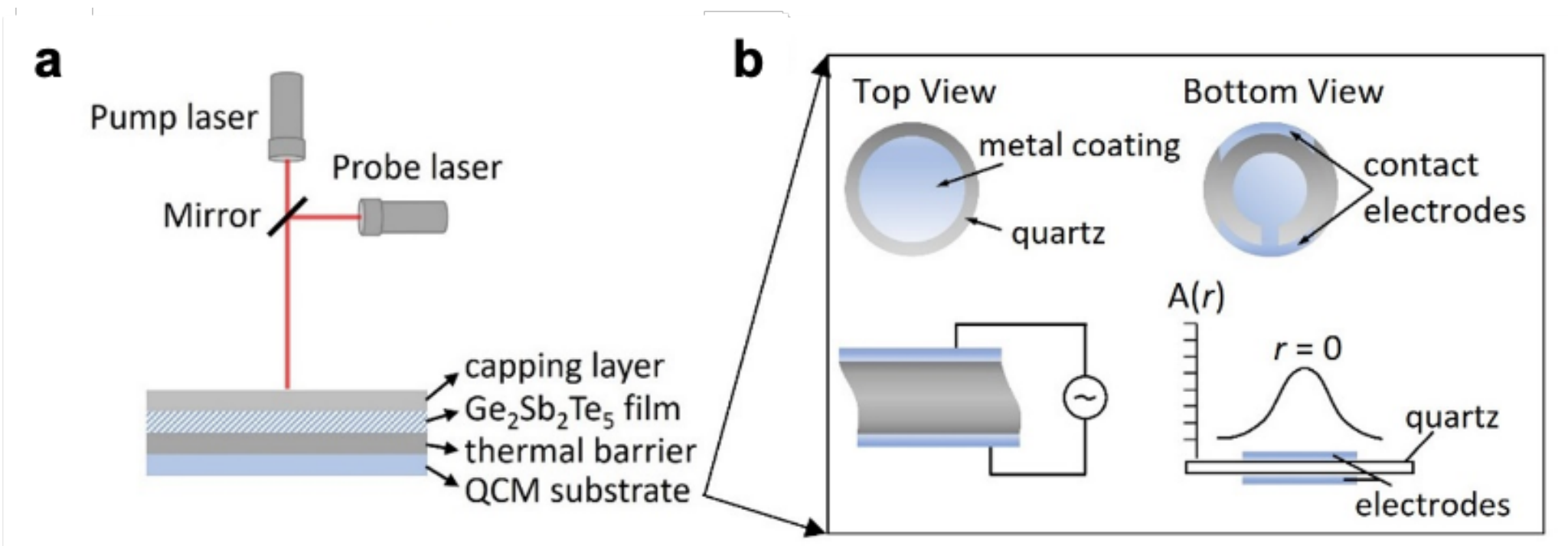

Supplementary Fig. 20. Experimental set-up. (**a**) The pump-probe laser set-up and an ultrasonic Quartz Crystal Microbalance (QCM) device. The GST thin film is sandwiched between a capping layer and a thermal barrier, which are deposited upon the QCM substrate. (**b**) Top and bottom view of the QCM device and the working mechanism of ultrasonic vibration generator QCM. External oscillator circuits drive the resonant and odd overtone shear vibration of a quartz crystal (see more details in Supplementary Sec.1.2).

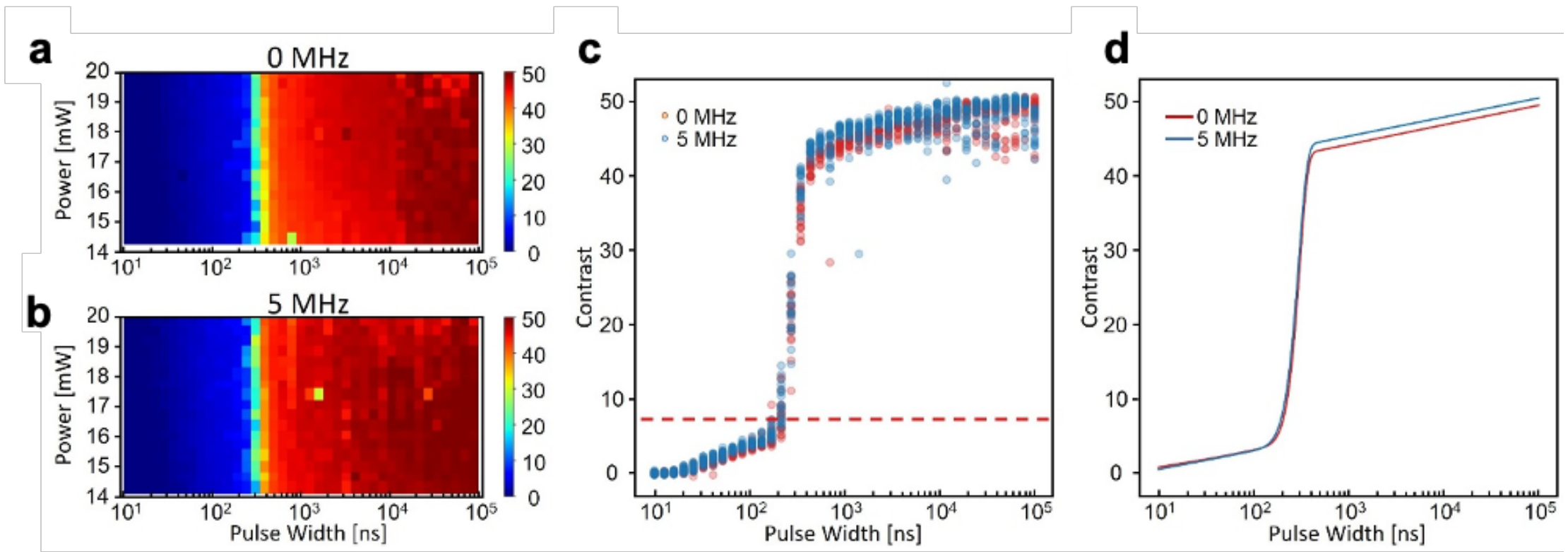

Supplementary Fig. 21. Fine-step PTE diagrams. Crystallization at ultrasonic vibrations at (**a**) 0MHz and (**b**) 5 MHz. The color scale represents the contrast and is set the same in both diagrams to reveal a higher contrast region in the 5 MHz plot. (**c**) Contrast at all laser power as a function of pulse width for 0 and 5 MHz. (**d**) Averaged contrast over laser power for 0 and 5 MHz.

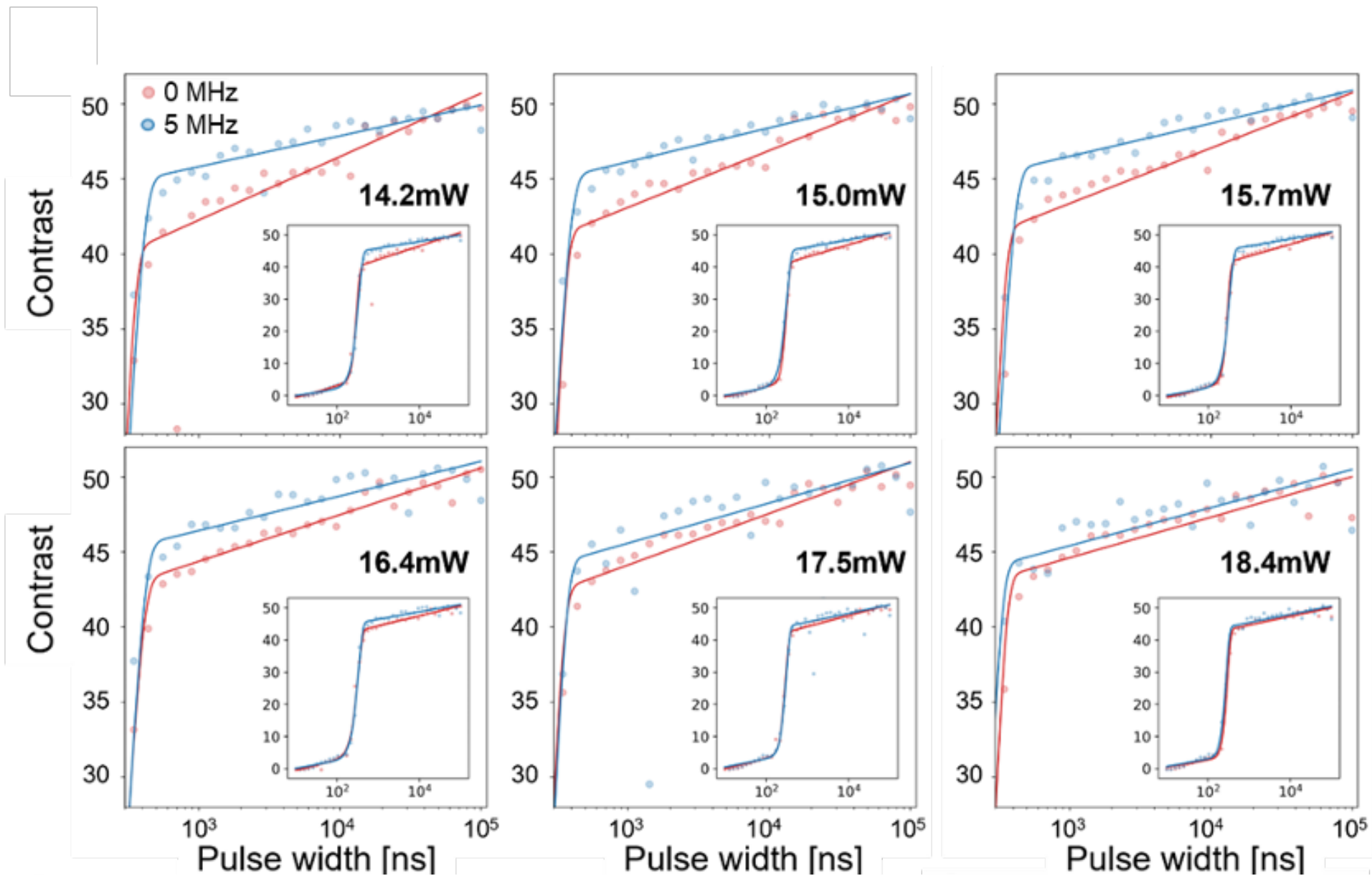

Supplementary Fig. 22. Contrast of the $Ge_2Sb_2Te_5$ film under varying laser power from the fine-step scans at 0 and 5 MHz.

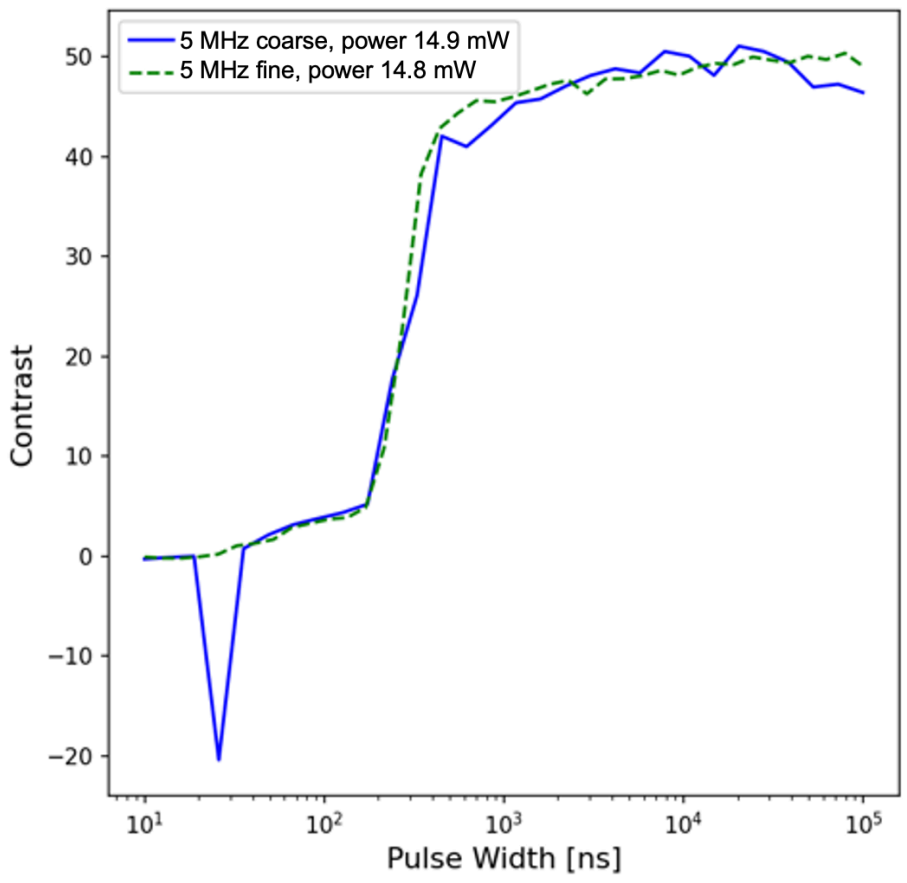

Supplementary Fig. 23. The contrast curves of the coarse- and fine-step scans at comparable laser power (14.9 mW for coarse and 14.8 mW for fine). The two measurements were carried out at different times and different locations of the film. The excellent overlap indicates a high homogeneity of the film and substrate and the absence of cumulative heating. The coarse curve has some jitters (possibly due to laser power fluctuations).

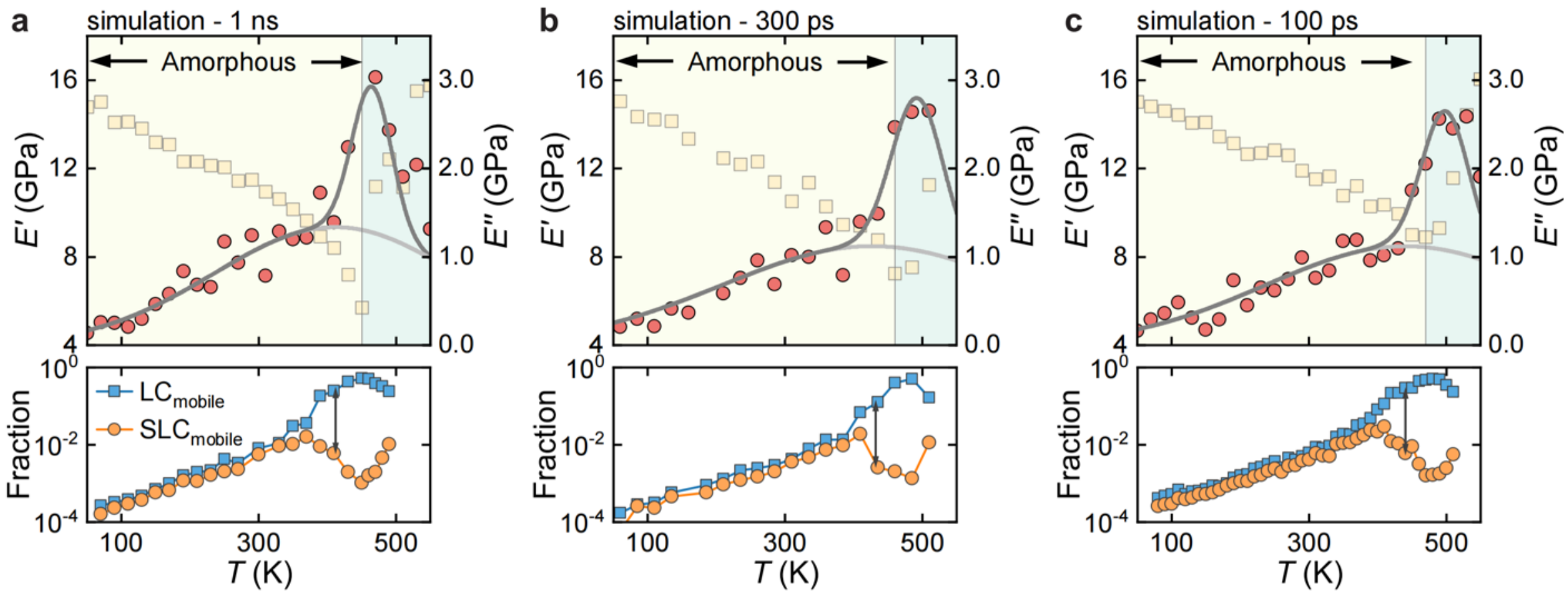

Supplementary Fig. 24. Frequency dependence of the β-relaxation and mobile-particle percolation in GST. (a–c) Results obtained from MD-DMS simulations with oscillation periods of 1 ns, 300 ps, and 100 ps, corresponding to frequencies of 1, 3.3, and 10 GHz, respectively. The upper panels show the temperature dependence of the storage modulus E′ (squares, left axis) and loss modulus E″ (circles, right axis). The lower panels show the fractions of atoms belonging to the largest mobile-particle cluster (LC_{mobile}, blue) and the second-largest mobile-particle cluster (SLC_{mobile}, orange). For each oscillation period, the β-relaxation temperature is determined from the mobile-particle percolation point, defined by $LC_{mobile}/SLC_{mobile} = 100$.

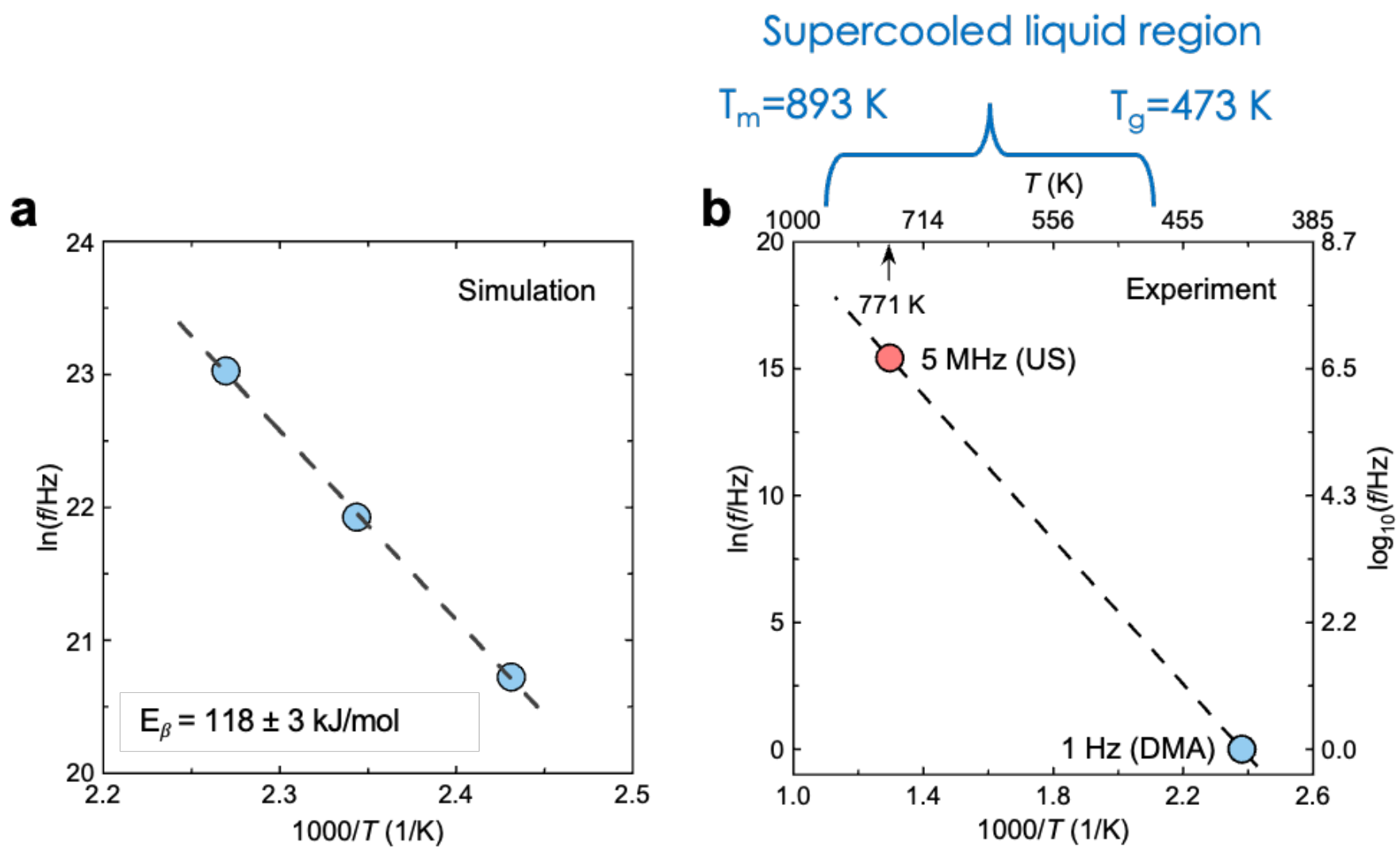

Supplementary Fig. 25. Arrhenius plots of β-relaxation frequency versus inverse temperature for GST. (a) Arrhenius fitting of the β-relaxation temperatures obtained from simulations at different frequencies, yielding an activation energy of 118 ± 3 kJ mol⁻¹. (b) Based on the activation energy obtained from the simulations, the experimentally measured β-relaxation at 1 Hz and 420 K is extrapolated to 5 MHz, corresponding to a characteristic temperature of approximately 771 K within the supercooled liquid region of GST.

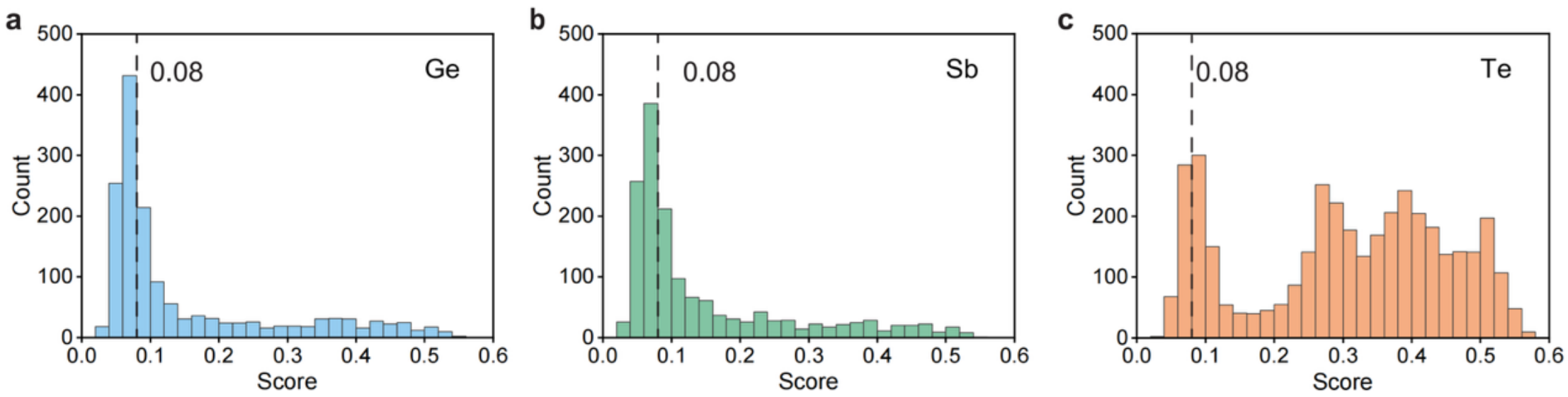

Supplementary Fig. 26. Distribution of cluster-alignment scores obtained in the template alignment mode for GST at 480 K. (a–c) correspond to Ge, Sb, and Te atoms, respectively. A smaller score indicates a better match with the crystalline template. The vertical dashed line marks the threshold score of 0.08 used to distinguish crystalline and amorphous atoms. This score is located around the first low-score peak of the score distributions, avoiding arbitrariness of choosing score.

References

1. Thompson AP, Aktulga HM, Berger R, Bolintineanu DS, Brown WM, Crozier PS, *et al.* LAMMPS - a flexible simulation tool for particle-based materials modeling at the atomic, meso, and continuum scales. *Computer Physics Communications* 2022, **271:** 108171.
2. Abou El Kheir O, Bonati L, Parrinello M, Bernasconi M. Unraveling the crystallization kinetics of the Ge2Sb2Te5 phase change compound with a machine-learned interatomic potential. *npj*

Computational Materials 2024, **10**(1)**:** 33.
3. Wang H, Zhang L, Han J, E W. DeePMD-kit: A deep learning package for many-body potential energy representation and molecular dynamics. *Computer Physics Communications* 2018, **228:** 178-184.
4. Alexandre A, Lavaud M, Fares N, Millan E, Louyer Y, Salez T*, et al.* Non-Gaussian Diffusion Near Surfaces. *Physical Review Letters* 2023, **130**(7)**:** 077101.
5. Xu H, Zheng X, Shi X. Deciphering non-Gaussianity of diffusion based on the evolution of diffusivity. *Physical Review Research* 2024, **6**(2)**:** 023014.
6. Rusciano F, Pastore R, Greco F. Fickian Non-Gaussian Diffusion in Glass-Forming Liquids. *Physical Review Letters* 2022, **128**(16)**:** 168001.
7. Larini L, Ottochian A, De Michele C, Leporini D. Universal scaling between structural relaxation and vibrational dynamics in glass-forming liquids and polymers. *Nature Physics* 2008, **4**(1)**:** 42-45.
8. Gao L, Sun Y, Yu H-B. Mobility percolation as a source of Johari-Goldstein relaxation in glasses. *Physical Review B* 2023, **108**(1)**:** 014201.
9. Gao L, Yu H-B, Schrøder TB, Dyre JC. Unified percolation scenario for the α and β processes in simple glass formers. *Nature Physics* 2025, **21**(3)**:** 471-479.
10. Ghosh A, Budrikis Z, Chikkadi V, Sellerio AL, Zapperi S, Schall P. Direct Observation of Percolation in the Yielding Transition of Colloidal Glasses. *Physical Review Letters* 2017, **118**(14)**:** 148001.
11. Fang XW, Wang CZ, Yao YX, Ding ZJ, Ho KM. Atomistic cluster alignment method for local order mining in liquids and glasses. *Physical Review B* 2010, **82**(18)**:** 184204.
12. Sun Y, Zhang F, Ye Z, Zhang Y, Fang X, Ding Z*, et al.* 'Crystal Genes' in Metallic Liquids and Glasses. *Scientific Reports* 2016, **6**(1)**:** 23734.
13. Battaglia, R. openQCM Q-1 User Manual. openQCM https://openqcm.com/shared/q-1/openQCM_Q-1-user_manual.pdf (2025)
14. Analog Devices, Inc. AD9851: CMOS 180 MHz DDS/DAC Synthesizer Data Sheet (Rev.D). Analog Devices https://www.analog.com/en/products/ad9851.html#documentation (1998).
15. Guha A, Sandström N, Ostanin V, Wijngaart W, Klenerman D, Ghosh S. Simple and ultrafast resonance frequency and dissipation shift measurements using a fixed frequency drive. *Sensors and Actuators B: Chemical* 2018, **281**.
16. Johannsmann, D. Modeling the resonator as a parallel plate. In *The Quartz Crystal Microbalance in Soft Matter Research: Fundamentals and Modeling* (ed. Johannsmann, D.), pp. 49–123 (Springer International Publishing, Cham, 2015). https://doi.org/10.1007/978-3-319-07836-6_4
17. Reviakine I, Johannsmann D, Richter RP. Hearing what you cannot see and visualizing what you hear: interpreting quartz crystal microbalance data from solvated interfaces. *Anal Chem* 2011, **83**(23)**:** 8838-8848.
